# On the prediction of tibiofemoral contact forces for healthy individuals and osteoarthritis patients during gait: a comparative study of regression methods

**DOI:** 10.1038/s41598-023-50481-x

**Published:** 2024-01-16

**Authors:** Felipe Arruda Moura, Alexandre R. M. Pelegrinelli, Danilo S. Catelli, Erik Kowalski, Mario Lamontagne, Ricardo da Silva Torres

**Affiliations:** 1 Laboratory of Applied Biomechanics, Sport Sciences Department, State University of Londrina, Londrina, Brazil; 2 Wageningen Data Competence Center, Wageningen University and Research, Wageningen, The Netherlands; 3 Human Movement Biomechanics Laboratory, University of Ottawa, Ottawa, Canada; 4 Department of Movement Sciences, Faculty of Movement and Rehabilitation Sciences, KU Leuven, Leuven, Belgium; 5 Department of ICT and Natural Sciences, NTNU-Norwegian University of Science and Technology, Ålesund, Norway

**Keywords:** Orthopaedics, Computer science, Laboratory techniques and procedures

## Abstract

Knee osteoarthritis (OA) is a public health problem affecting millions of people worldwide. The intensity of the tibiofemoral contact forces is related to cartilage degeneration, and so is the importance of quantifying joint loads during daily activities. Although simulation with musculoskeletal models has been used to calculate joint loads, it demands high-cost equipment and a very time-consuming process. This study aimed to evaluate consolidated machine learning algorithms to predict tibiofemoral forces during gait analysis of healthy individuals and knee OA patients. Also, we evaluated three different datasets to train each model, considering different combinations of primary kinematic and kinetic data, and post-processing data. We evaluated 14 patients with severe unilateral knee OA and 14 healthy individuals during 3–5 gait trials. Data were split into 70% and 30% of the samples as training and test data. Test data was independently evaluated considering a mixture of pathological and healthy individuals, and only OA and Control patients. The main results showed that accurate predictions of the tibiofemoral contact forces were achieved using machine learning methods and that the predictions were sensitive to changes in the input data as training. The present study provided insights into the most promising regressions methods to predict knee contact forces representing an important starting point for the broader application of biomechanical analysis in clinical environments.

## Introduction

The prevalence of knee osteoarthritis (OA) is increasing worldwide. In the United States, for example, knee OA affects 12% of those above 60 years old and has a significant economic impact on health systems, with a cost of approximately USD 140,000 per patient over their lifetime^1–3^. Strong evidence considers that structural and mechanical changes in joints are responsible for the development and progression of OA^4,5^. The intensity and distribution of forces in knee regions throughout life are related to articular cartilage degeneration^6–8^.

During the gait stance phase, the resultant tibiofemoral contact force presents a waveform with two clear peaks, the first as a result of initial contact of the foot with the ground during the loading response sub-phase and the second relative to the propulsion of the body forward during gait^9,10^. Considering the intensity and distribution of forces on the knee as one of those responsible for the onset and progression of knee OA, these forces must be analyzed. For a non-invasive analysis of joint contact forces, musculoskeletal (MSK) modelling approaches have been used^10,11^. MSK modeling platforms extend the utility of biomechanics lab measurement by coupling joint kinematics and ground reaction forces (GRFs) with computational methods to estimate muscle and joint reaction forces during human movements. With the development of theoretical and experimental methods to improve accuracy and reliability, human motion analysis has become a useful investigative and diagnostic tool in many research and clinical areas, such as medicine, ergonomics, and sports^12^.

Quantitative analysis in biomechanics generally requires a set of equipment, synchronization devices, and consumable materials. In practice, that means substantial investments are needed, considering typical technologies commercially available to support data acquisition. Thus, this demand restricts the exploration and application of biomechanics analysis in minor clinical settings, hospitals in underdeveloped countries, schools, sports clubs, etc. A suitable alternative relies on non-expensive (semi)-automatic methods. Machine learning (ML) is a class of algorithms frequently used for making predictions based on statistical patterns discovered from data. Several studies have applied these data-driven algorithms to gait lab prediction tasks to avoid hardware or computational bottlenecks by exploiting the inference capabilities of trained machine learning models^13–16^. Although some relevant research has been conducted with machine learning algorithms to predict discrete and time series kinematic and kinetic data for gait analysis, generalized models applied to healthy and pathological participants is underreported.

To address this issue, this study assessed the prediction of gait tibiofemoral contact forces of healthy and OA individuals followed by various regression techniques (24 different algorithms in total). We also adjudge the performance of each regression technique considering different sets of predictors, i.e., we evaluated if only primary kinematic (joint angles) and kinetic data (ground reaction forces) are enough to outcome accurate predictions, and how is the influence of including post-processing data, such as joint moments and muscles forces. We hypothesized that it is possible to accurately predict tibiofemoral contact forces of both healthy and pathological individuals from primary kinetic and kinematic data using ML methods. However, better performance would be achieved when post-processing data is presented.

## Background

Biomechanics is the science that examines forces acting upon and within a biological structure and the effects produced by such forces. Biomechanics has an interdisciplinary approach and several investigation methods that provide information about the internal and external mechanics associated with the locomotion^17^.

Kinematic information obtained from the quantitative analysis provides data from the body and segments’ position, orientation, velocity, and acceleration. Combined with kinetic and segment parameters data, information relative to the center of mass, segment energy levels and power, joint moments and forces can be computed^18^. Joint kinematics and ground reaction forces (GRFs) offer measurable quantities that characterize movement quality and form the basis of a biomechanics laboratory assessment.

MSK models have been used for a non-invasive analysis of joint contact forces. When using MSK models, the choice of the model is critical, considering the variables of interest and the capacity of the model to estimate the contact forces^19^. However, patient kinematics and mechanics derived from MSK requires a multi-stage computational pipeline, including subject-specific calibration and scaling, as well as manual optimization procedures^13^. In this sense, ML algorithms are a suitable alternative to predict muscle and joint reaction forces.

Burton and colleagues^13^ evaluated four different ML algorithms to estimate joint contact and muscle forces activities of daily living based on anthropometric, GRFs, and joint angle data of total knee arthroplasty (TKA) patients. Patient mechanics were accurately predicted by recurrent neural networks, even considering fewer predictor variables. A similar approach was conducted by Giarmatzis et al.^14^ with young and elderly participants during treadmill walking. The authors assessed artificial neural networks (ANNs) and support vector regression (SVR) algorithms based on kinematics data and considered the inclusion/exclusion of GRFs in the dataset during training steps. ANNs presented the best-performing predictor of knee contact forces and excluding GRFs data did not substantially decrease the prediction power. Also, using ANNs, healthy participants’ knee flexion and adduction moments during various locomotion tasks were predicted in the study of Stetter and colleagues^20^. Recent research^15^ showed promising results for Random Forest (RF) and Convolutional Neural Networks (CNN) algorithms to predict kinematics and kinetics outcomes from inertial measurement unit (IMU) data of healthy individuals during walk trials. When pathological conditions were evaluated, Aljaaf and colleagues^21^ successfully predicted the frontal plane internal knee abduction moment of patients with alkaptonuria. From kinematics data, the authors evaluated four ML algorithms: Decision Tree, Random Forest, Linear Regression, and Multilayer Perceptron neural network. The Multilayer Perceptron neural network method presented superior results, considering both algorithms’ performance and speed. Also, in a previous study^22^, knee contact force was accurately predicted by integrating the Artificial Fish Swarm and the Random Forest algorithm. However, the authors evaluated data of only three patients implanted with an instrumented knee replacement, requiring evaluation of a possible generalization of the algorithms for a larger variability dataset.

Relative to knee OA patients, a previous study^23^ considered almost 500 participants. Personal cameras were utilized to record a 5-trial sit-to-stand task. Later, participants were invited to answer a survey, including physical and mental health characteristics, and OA status. The authors reported that the trunk kinematics parameters are sensitive enough to predict physical health and OA. A recent study^24^ applied the probabilistic principal component analysis (PPCA) model in IMU data of knee OA patients to predict tibiofemoral contact forces during gait. The root mean square error ranged from 0.15 to 0.40 of body weight, with moderate to strong correlations between contact forces estimated by MSK and PPCA models. Finally, the feasibility of using IMU training data from people with knee OA performing multiple clinically important activities was evaluated to predict knee joint sagittal plane kinematics using a deep learning approach^25^. However, none of these studies dealt with predicting joint reaction forces in knee OA patients and healthy individuals. One can argue that generic models may not properly predict the biomechanical data of pathological groups, and vice-versa.

Relevant research provided insights regarding using machine learning algorithms to address classification and prediction tasks involving biomechanical data. In the present study, we advance the state of the art regarding exploring of a broader set of ML techniques and their parameter settings, to predict tibiofemoral contact forces for both healthy individuals and OA patients. To the best of our knowledge, this is the first study to explore such a range of techniques and the first with knee OA patients. We also proposed to investigate the accuracy of different combinations of discrete data to predict the first and the second tibiofemoral contact force peaks during the gait stance phase. For a comprehensive evaluation with a clinical focus, we trained the models using data from healthy individuals and OA patients. However, separate tests were also conducted to ensure accuracy.

## Materials and methods

### Participants

The study evaluated 14 individuals with severe unilateral knee OA (KL4)^26^. The group included six females and eight males, with a median age of 63.7 (55.2; 68.1) years old, 1.67 m (1.61; 1.77) height and 80.2 kg (70.4; 85.3) weight. For the control groups, 14 healthy individuals were evaluated, seven females and seven males, with 63 (60; 64) years old, 1.69 m (1.63; 1.73) height, and 73.6 kg (61.0; 77.7) weight. Participants with body mass index (BMI) higher than 35 kg/m$^2$ and a waist circumference higher than 102 cm for males and 88 cm for females were excluded from both groups. In the OA group, participants who had undergone any joint replacement for lower limbs or with any other degenerative joint conditions than the affected knee were excluded, as well, for both groups, any other conditions that could affect the gait.

The University of Ottawa and the Ottawa Hospital Research Institute ethics committees approved the study. All participants provided written informed consent, and the research was conducted by the principles of good clinical practice and the Declaration of Helsinki.

### Data collection

The data collection was performed with ten infrared cameras (200 Hz, 2 Vantage V5 and 8 Vero 2.2, Vicon, Oxford Metrics, UK) and four force plates (1000 Hz, model 9286B, Kistler; model FP4060, Bertec, USA) embedded in the floor, in the middle distance of the ten meters walkway. For tracking the segments, the University of Ottawa Motion Analysis Model (UOMAM) marker set was used^27^. A static kinematic capture was performed in a similar anatomical position with shoulder abduction of around 30 degrees. Next, three to five gait trials were performed at a self-selected pace.

### Data processing

**Figure 1:**
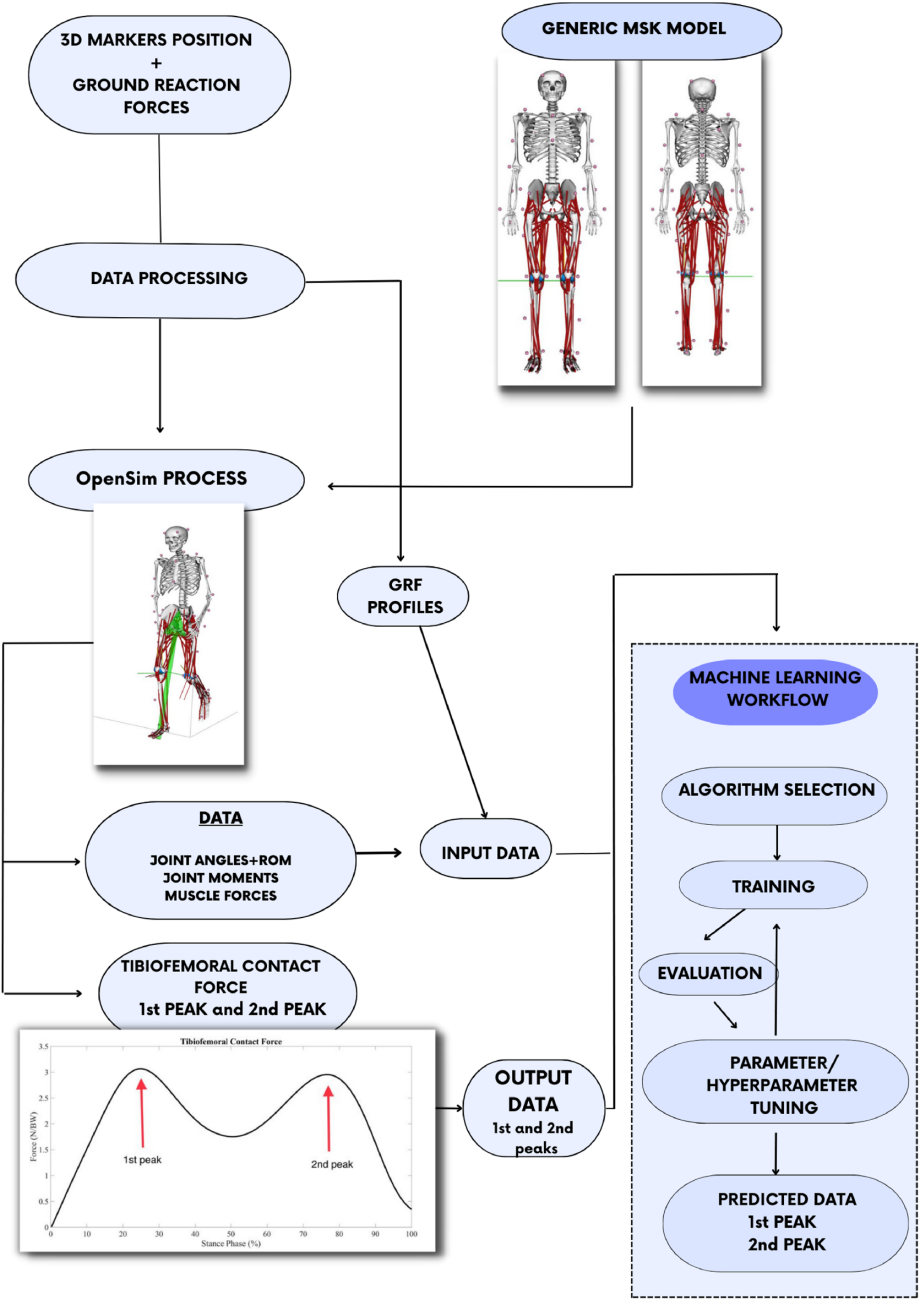
The methodology workflow, from data collection to machine learning setup for tibiofemoral contact forces prediction.

The workflow of the study methodology is presented in Fig. 1. First, the marker trajectories were labeled using the manufacturer’s software, and the gaps were filled. The force plate data were filtered with a 4th order (zero lag) Butterworth filter with a cut-off frequency of 10 Hz. A Woltring filter with a mean standard error of 15 mm was applied for the kinematic data. The gait stance phase was cropped using the foot strike and the foot off using the vertical force signal from the force plate, with a threshold of 10 N. The stance phase was normalized to 101 points, and then data was exported for OpenSim formats.

Using the OpenSim 3.3 software^28^, a generic model was scaled using a marker-based approach. The generic MSK model employed^29^ implemented muscle parameters that reduced late-stance knee contact force^30^. Basically, the adjustments were: (a) knee mediolateral translation was locked, (b) adjustments in passive muscle forces and tendon compliance proposed by Uhlrich et al.^30^ , and (c) muscle-tendon units paths for gluteus medius, gluteus minimus, and tensor fascia latae were adjusted about the origin (moved superiorly and laterally) and insertion (anteriorly). The MSK model developed in OpenSim and employed in this study is available for download (see Sect. 7). The model included 80 lower-limb Hill-type muscle-tendon units with 37 degrees of freedom and 17 ideal torque actuators driving the upper body^31^. The model allowed for estimating the medial and lateral compartments of the vertical tibiofemoral contact force^9,32^.

The inverse kinematics, inverse dynamics, static optimization, and joint reaction analyses (JRA) were processed using the Batch OpenSim Processing Scripts (BOPS) Matlab toolbox^33^. Static optimization was employed to calculate the muscle activation and forces, which minimized the sum of squared muscle activation^11^. The JRA computed the resultant forces and moments in each joint. For tibiofemoral forces, the total force was considered as the sum of the lateral and medial compartment vertical forces^32^. Thus, the time series for all variables were extracted as a function of the stance phase.

### Dataset organization and machine learning algorithms

Considering that all participants ($n = 28$) performed 3–5 trials, the data source was formed by 126 elements. Data were split into 90 samples for training data (70%) and 36 for test data (30%), according to recent recommendations regarding optimal ratio for data splitting^34^. Samples related to a single participant were included either in the training set or in the test set, i.e., no participants from the training dataset were included in the test dataset. The test data was further independently evaluated into three forms: All Participants (36 samples), OA Patients (20 samples), and Control Individuals (16 samples).

To evaluate the dependency between the predicting variables and the accuracy of the tibiofemoral contact forces, three input datasets were assessed (Table 1):

- Input 1: only with primary kinematic and kinetic data;
- Input 2: data from Input 1 with hip and knee moments; and
- Input 3: data from Input 2 with muscle forces.

**Table 1:** Dataset input assessed by the machine learning algorithms.

Dataset	Predictors	Input 1	Input 2	Input 3
Kinetic primary data	Vertical GRF (1st peak)	$\checkmark$	$\checkmark$	$\checkmark$
	Vertical GRF (2nd peak)	$\checkmark$	$\checkmark$	$\checkmark$
	Vertical GRF (curve integral)	$\checkmark$	$\checkmark$	$\checkmark$
	Anteroposterior GRF (peak value)	$\checkmark$	$\checkmark$	$\checkmark$
	Anteroposterior GRF (minimum value)	$\checkmark$	$\checkmark$	$\checkmark$
	Mediolateral GRF (1st peak)	$\checkmark$	$\checkmark$	$\checkmark$
	Mediolateral GRF (2nd peak)	$\checkmark$	$\checkmark$	$\checkmark$
Kinematic primary data	Hip: peak flexion angle	$\checkmark$	$\checkmark$	$\checkmark$
	Hip: peak extension angle	$\checkmark$	$\checkmark$	$\checkmark$
	Hip: flexion/extension ROM	$\checkmark$	$\checkmark$	$\checkmark$
	Hip: peak abduction angle	$\checkmark$	$\checkmark$	$\checkmark$
	Hip: peak adduction angle	$\checkmark$	$\checkmark$	$\checkmark$
	Hip: abduction/adduction ROM	$\checkmark$	$\checkmark$	$\checkmark$
	Knee: peak flexion angle	$\checkmark$	$\checkmark$	$\checkmark$
	Knee: peak extension angle	$\checkmark$	$\checkmark$	$\checkmark$
	Knee: flexion/extension ROM	$\checkmark$	$\checkmark$	$\checkmark$
Joint Moments	Hip: peak flexion moment	–	$\checkmark$	$\checkmark$
	Hip: peak extension moment	–	$\checkmark$	$\checkmark$
	Hip: abduction moment (1st peak)	–	$\checkmark$	$\checkmark$
	Hip: abduction moment (2nd peak)	–	$\checkmark$	$\checkmark$
	Knee: peak flexion moment	–	$\checkmark$	$\checkmark$
	Knee: peak extension moment	–	$\checkmark$	$\checkmark$
Muscle forces	Vastus medialis peak	–	–	$\checkmark$
	Vastus medialis curve integral	–	–	$\checkmark$
	Vastus lateralis peak	–	–	$\checkmark$
	Vastus lateralis curve integral	–	–	$\checkmark$
	Vastus intermedius peak	–	–	$\checkmark$
	Vastus intermedius curve integral	–	–	$\checkmark$
	Rectus femoris first peak	–	–	$\checkmark$
	Rectus femoris second peak	–	–	$\checkmark$
	Rectus femoris curve integral	–	–	$\checkmark$
	Biceps femoris (long head) peak	–	–	$\checkmark$
	Biceps femoris (long head) curve integral	–	–	$\checkmark$
	Biceps femoris (short head) peak	–	–	$\checkmark$
	Biceps femoris (short head) curve integral	–	–	$\checkmark$
	Semimembranosus peak	–	–	$\checkmark$
	Semimembranosus curve integral	–	–	$\checkmark$
	Semitendinosus peak	–	–	$\checkmark$
	Semitendinosus curve integral	–	–	$\checkmark$
	Gastrocnemius medialis peak	–	–	$\checkmark$
	Gastrocnemius medialis integral	–	–	$\checkmark$
	Gastrocnemius lateralis peak	–	–	$\checkmark$
	Gastrocnemius lateralis integral	–	–	$\checkmark$
	Hip abductors first peak	–	–	$\checkmark$
	Hip abductors second peak	–	–	$\checkmark$
	Hip abductors curve integral	–	–	$\checkmark$
	Gluteus medius first peak	–	–	$\checkmark$
	Gluteus medius second peak	–	–	$\checkmark$
	Gluteus medius curve integral	–	–	$\checkmark$

In total, 24 machine learning algorithms were evaluated in the present study. Several experiments were performed for each algorithm to identify the best parameters based on training accuracy. The algorithms were selected based on previous literature with biomechanics and health sciences data prediction^13,14,23,25,35^. A brief description of the parameters and hyperparameters tuning (when applicable) tested and selected over experiments and respective references for each algorithm are presented in Table 12 in Appendix A.

### Model evaluation and statistical analysis

The performance of each model for training and each test dataset (All Participants, OA Patients, and Control Individuals) considering the three input options (Input 1, Input 2, and Input 3) was evaluated based on mean absolute error (MAE), root mean squared error (RMSE), Mean Delta Force (i.e., the difference between MSK model tibiofemoral force and predicted tibial force) and 95% Confidence Interval (CI), Pearson Correlation Coefficient (R), the coefficient of determination (R$^2$). The coefficient of determination R$^2>0.70$ was defined as high^36,37^. Additionally, to have a measure of the error relative to the peak values estimated by the MSK model, we calculated the relative peak error:

1 $$\begin{aligned} RPE = \frac{ \left| Predicted_{Peak}-MSK_{Peak} \right| }{MSK_{Peak}}\times 100 \end{aligned}$$

All the algorithms and performance analyses were run using Matlab Software (MATLAB R2021b—MathWorks, Inc., Natick, MA, USA). Part of the algorithms was personally written based on previous literature codes^35^. The parameters/hyperparameters tested for each model, the training and independent tests steps were performed in an Intel$^{(R)}$ Core$^{(TM)}$ i7-9750H generation and NVIDIA GeForce RTX 2060 GPU machine.

## Results

**Figure 2:**
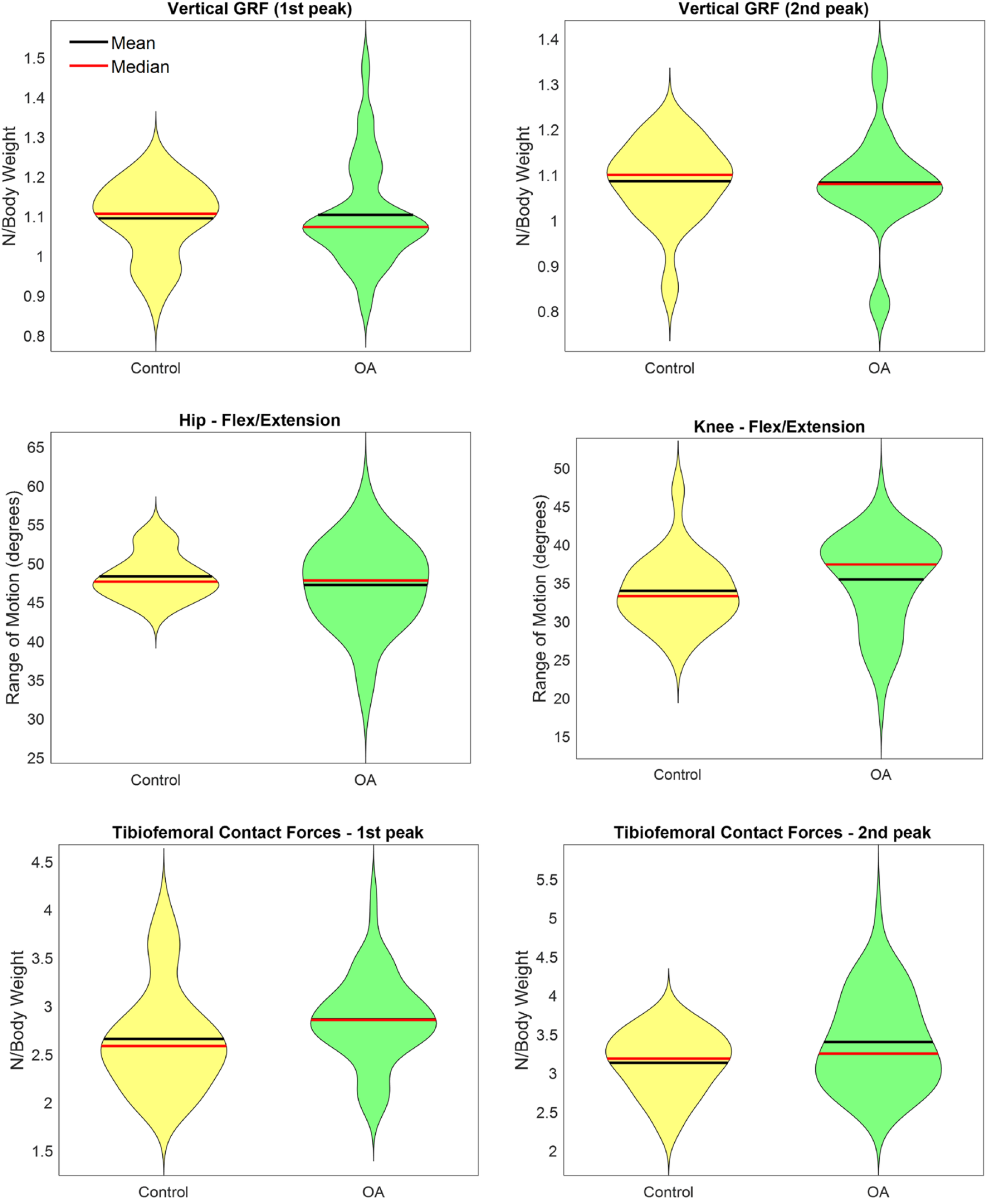
Descriptive statistics of selected data explored in the present study for both control and osteoarthritis groups.

Figure 2 presents, for both OA and Control groups, violin plots of selected kinetic and kinematic data used as predictors variables, as well as the predicted variables (1st and 2nd tibiofemoral force peaks) during gait. At the top, the vertical ground reaction forces peaks during gait were represented. At the center of the figure, kinematic data was exemplified by the hip and knee range of motion at the sagittal plane. At the bottom, the key-predicted variables of the present study were represented, calculated from the musculoskeletal model.

Tables 13, 14 and 15 in Appendix B present the training results for each model, considering Inputs 1, 2, and 3, respectively. As expected, most of the ML models presented high coefficients of determination and low errors.

The experimental results on independent tests were performed considering three groups: all participants (formed by healthy individuals and knee osteoarthritis patients), OA patients, and Controls (formed only by healthy participants). Tables 2, 3, and 4 present the performance of each model for the All Participants group, considering Inputs 1, 2, and 3 as training data, respectively.

When Input 1 was applied as training data, the range of MAE for the 1st peak ranged from 0.17 to 0.49. The Gaussian Regression (Kernel-exponential) presented the highest accuracy (in bold lettering), but good performance was identified for Gaussian Regression (Kernel-matern 32) and Gaussian SVR. For the prediction of the 2nd peak, results presented lower accuracy with MAE ranging from 0.28 to 0.91. The higher accuracy was achieved by the DNNE model (in bold text). When Input 2 was set as training data, MAE ranged from 0.19 to 0.68, with higher accuracy found for Gaussian Regression (Kernel-matern 32). For the 2nd peak, poor results were found, with MAE ranging from 0.29 to 0.75.

Interestingly, for both peaks, proving more information (i.e., Input 2 considers data from Input 1 and joint moments data) as training data did not provide increased accuracy. However, when Input 3 was used as training data, increased performance was identified. For the 1st peak, MAE ranged from 0.09 to 0.67. The Gaussian SVR model achieved the highest accuracy, but promising results were also identified for Gaussian Regression (Kernel-matern 32 and 52). Considering the predictions of the 2nd peak, MAE ranged from 0.16 to 0.55, with higher accuracy found for the Linear SVR model.

**Table 2:** Summary of the performance of the algorithms for all participants group, considering Input 1 as training data.

Function	1st Knee contact peak (N/body weight)								2nd Knee contact peak (N/body weight)							
	MAE	RPE	RMSE	R	R2	MDF	LCI	UCI	MAE	RPE	RMSE	R	R2	MDF	LCI	UCI
(1) Ensemble trees (bagging)	0.25	8.67	0.32	0.64	0.41	$-$ 0.15	$-$ 0.25	$-$ 0.06	0.46	13.14	0.63	0.15	0.02	$-$ 0.25	$-$ 0.44	$-$ 0.05
(2) Ensemble trees (LSBoost)	0.36	12.59	0.42	0.49	0.24	$-$ 0.02	$-$ 0.16	0.13	0.46	14.09	0.63	0.34	0.11	$-$ 0.09	$-$ 0.30	0.13
(3) Linear SVR	0.29	10.32	0.35	0.78	0.61	0.23	0.14	0.32	0.58	17.69	0.69	0.16	0.02	$-$ 0.18	$-$ 0.41	0.05
(4) Quadratic SVR	0.39	13.60	0.51	0.44	0.19	$-$ 0.27	$-$ 0.42	$-$ 0.13	0.59	17.81	0.69	0.01	0.00	$-$ 0.14	$-$ 0.38	0.09
(5) Cubic SVR	0.32	11.20	0.39	0.55	0.30	$-$ 0.24	$-$ 0.35	$-$ 0.13	0.91	30.08	1.49	0.36	0.13	$-$ 0.48	$-$ 0.96	0.01
(6) Gaussian SVR	0.19	6.79	0.23	0.88	0.77	$-$ 0.16	$-$ 0.21	$-$ 0.11	0.48	14.68	0.53	0.02	0.00	0.04	$-$ 0.14	0.22
(7) Linear regression	0.20	7.43	0.25	0.77	0.59	0.13	0.05	0.20	0.59	17.49	0.75	0.25	0.07	$-$ 0.21	$-$ 0.46	0.03
(8) Lasso regression	0.21	7.74	0.27	0.71	0.50	0.10	0.02	0.19	0.58	17.26	0.70	0.07	0.01	$-$ 0.20	$-$ 0.43	0.03
(9) Ridge regression	0.28	10.60	0.39	0.59	0.35	0.19	0.08	0.31	0.60	18.40	0.69	0.06	0.00	$-$ 0.09	$-$ 0.32	0.14
(10) Binary decision tree	0.31	11.06	0.39	0.64	0.41	$-$ 0.24	$-$ 0.34	$-$ 0.13	0.48	14.24	0.58	0.14	0.02	$-$ 0.09	$-$ 0.28	0.11
(11) GR (K.-exponential)	**0.17**	**5.94**	**0.21**	**0.88**	**0.77**	$-$ ** 0.12**	$-$ **0.18**	$-$ **0.07**	0.47	14.10	0.56	0.19	0.04	$-$ 0.02	$-$ 0.22	0.17
(12) GR (K.-squared exponential)	0.22	7.68	0.25	0.86	0.74	$-$ 0.19	$-$ 0.25	$-$ 0.13	0.46	13.97	0.52	0.05	0.00	0.03	$-$ 0.15	0.20
(13) GR (K.-matern 32)	0.17	6.58	0.22	0.75	0.56	$-$ 0.01	$-$ 0.09	0.06	0.47	14.41	0.54	0.10	0.01	0.01	$-$ 0.17	0.20
(14) GR (K.-matern 52)	0.20	7.06	0.23	0.89	0.79	$-$ 0.17	$-$ 0.23	$-$ 0.12	0.47	14.36	0.53	0.06	0.00	0.02	$-$ 0.16	0.20
(15) GR (K.-rational quadratic)	0.20	6.94	0.23	0.89	0.79	$-$ 0.17	$-$ 0.22	$-$ 0.12	0.46	14.17	0.53	0.02	0.00	0.02	$-$ 0.16	0.20
(16) ETSVR-Kernel linear	0.35	12.62	0.47	0.63	0.40	0.26	0.12	0.39	0.57	17.49	0.69	0.05	0.00	$-$ 0.17	$-$ 0.40	0.06
(17) Kernel ridge regression	0.37	13.16	0.49	0.65	0.42	0.28	0.14	0.41	0.62	19.04	0.72	0.00	0.00	$-$ 0.10	$-$ 0.35	0.14
(18) Nyström Ridge Regression	0.44	15.53	0.60	0.61	0.37	0.34	0.17	0.51	0.62	19.02	0.71	0.02	0.00	$-$ 0.09	$-$ 0.33	0.16
(19) DNNE	0.41	14.58	0.49	0.73	0.54	$-$ 0.07	$-$ 0.23	0.10	**0.28**	**9.05**	**0.38**	**0.68**	**0.46**	**0.03**	$-$ **0.10**	**0.16**
(20) kNN weighted mean	0.49	16.93	0.58	0.53	0.28	$-$ 0.49	$-$ 0.60	$-$ 0.38	0.46	13.23	0.61	0.02	0.00	$-$ 0.25	$-$ 0.44	$-$ 0.06
(21) RKNNWTSVR	0.31	11.39	0.42	0.68	0.46	0.23	0.11	0.35	0.57	17.25	0.70	0.03	0.00	$-$ 0.21	$-$ 0.43	0.02
(22) LTSVR	0.38	13.74	0.53	0.55	0.31	0.28	0.13	0.44	0.56	17.46	0.65	0.12	0.01	$-$ 0.05	$-$ 0.28	0.17
(23) Stepwise glm	0.21	7.57	0.26	0.66	0.44	$-$ 0.06	$-$ 0.15	0.02	0.48	14.46	0.60	0.24	0.06	$-$ 0.21	$-$ 0.40	$-$ 0.02
(24) Neural networks	0.25	9.24	0.30	0.68	0.46	0.03	$-$ 0.08	0.13	0.76	22.45	0.92	0.48	0.23	$-$ 0.11	$-$ 0.43	0.20

Best results (i.e. highest accuracy) are in bold.

*RMSE* root mean squared error, *R* Pearson correlation coefficient, (*R*$^2$) the coefficient of determination, *MDF* mean delta force, *LCI* lower confidence interval, *UPF* upper confidence interval, *GR* Gaussian regression, *K* Kernel.

**Table 3:** Summary of the performance of the algorithms for all participants group, considering input 2 as training data.

Function	1st Knee contact peak (N/body weight)								2nd Knee contact peak (N/body weight)							
	MAE	RPE	RMSE	R	R2	MDF	LCI	UCI	MAE	RPE	RMSE	R	R2	MDF	LCI	UCI
(1) Ensemble trees (bagging)	0.27	9.59	0.35	0.58	0.33	0.03	$-$ 0.09	0.15	0.45	12.65	0.61	0.10	0.01	$-$ 0.24	$-$ 0.44	$-$ 0.05
(2) Ensemble trees (LSBoost)	0.33	11.29	0.41	0.45	0.20	$-$ 0.05	$-$ 0.19	0.09	0.61	19.17	0.76	0.16	0.03	$-$ 0.17	$-$ 0.42	0.09
(3) Linear SVR	0.29	10.29	0.35	0.78	0.60	0.23	0.13	0.32	0.37	11.11	0.47	0.39	0.15	$-$ 0.05	$-$ 0.21	0.11
(4) Quadratic SVR	0.29	10.17	0.37	0.64	0.41	$-$ 0.05	$-$ 0.18	0.07	0.29	10.04	0.40	0.40	0.16	$-$ 0.20	$-$ 0.32	$-$ 0.08
(5) Cubic SVR	0.22	7.80	0.29	0.53	0.28	$-$ 0.06	$-$ 0.16	0.04	0.52	15.25	0.67	0.08	0.01	$-$ 0.28	$-$ 0.49	$-$ 0.07
(6) Gaussian SVR	0.19	6.85	0.22	0.87	0.76	$-$ 0.15	$-$ 0.20	$-$ 0.09	0.49	15.28	0.57	0.06	0.00	0.08	$-$ 0.12	0.27
(7) Linear regression	0.20	7.37	0.25	0.77	0.59	0.12	0.05	0.20	0.53	15.39	0.68	0.28	0.08	$-$ 0.16	$-$ 0.38	0.07
(8) Lasso regression	0.20	7.09	0.24	0.72	0.51	0.06	$-$ 0.02	0.14	0.62	17.98	0.79	0.32	0.10	$-$ 0.23	$-$ 0.49	0.03
(9) Ridge regression	0.27	9.76	0.37	0.58	0.34	0.15	0.03	0.26	0.59	17.45	0.72	0.28	0.08	$-$ 0.02	$-$ 0.26	0.23
(10) Binary decision tree	0.46	15.84	0.60	0.41	0.16	0.11	$-$ 0.10	0.31	0.55	16.37	0.68	0.28	0.08	$-$ 0.09	$-$ 0.32	0.15
(11) GR (K.-exponential)	0.22	7.68	0.25	0.86	0.74	$-$ 0.19	$-$ 0.25	$-$ 0.13	0.46	14.08	0.53	0.09	0.01	$-$ 0.05	$-$ 0.23	0.13
(12) GR (K.-squared exponential)	0.19	6.76	0.24	0.85	0.72	$-$ 0.12	$-$ 0.19	$-$ 0.05	0.42	12.75	0.52	0.09	0.01	0.00	$-$ 0.17	0.18
(13) GR (K.-matern 32)	**0.19**	**6.64**	**0.22**	**0.89**	**0.79**	$-$ **0.16**	$-$ **0.21**	$-$ **0.11**	0.48	14.30	0.59	0.22	0.05	$-$ 0.08	$-$ 0.28	0.12
(14) GR (K.-matern 52)	0.20	7.06	0.23	0.89	0.79	$-$ 0.17	$-$ 0.23	$-$ 0.12	0.48	14.36	0.56	0.07	0.01	$-$ 0.08	$-$ 0.27	0.11
(15) GR (K.-rational quadratic)	0.20	6.94	0.23	0.89	0.79	$-$ 0.17	$-$ 0.22	$-$ 0.12	0.47	14.12	0.54	0.06	0.00	$-$ 0.06	$-$ 0.24	0.13
(16) ETSVR-Kernel linear	0.32	11.29	0.44	0.57	0.32	0.21	0.07	0.34	0.47	14.13	0.58	0.06	0.00	0.02	$-$ 0.18	0.21
(17) Kernel ridge regression	0.37	12.95	0.54	0.55	0.31	0.24	0.07	0.40	0.51	15.42	0.62	0.07	0.01	0.02	$-$ 0.19	0.24
(18) Nyström ridge regression	0.68	23.44	0.90	0.23	0.05	$-$ 0.12	$-$ 0.43	0.18	0.75	22.06	0.89	0.44	0.19	$-$ 0.04	$-$ 0.35	0.26
(19) DNNE	0.44	15.34	0.53	0.71	0.50	$-$ 0.02	$-$ 0.20	0.16	0.70	22.09	0.85	0.33	0.11	$-$ 0.32	$-$ 0.59	$-$ 0.05
(20) kNN weighted Mean	0.49	16.93	0.58	0.53	0.28	$-$ 0.49	$-$ 0.60	$-$ 0.38	0.46	13.23	0.61	0.02	0.00	$-$ 0.25	$-$ 0.44	$-$ 0.06
(21) RKNNWTSVR	0.28	10.28	0.39	0.67	0.45	0.20	0.09	0.32	0.42	12.32	0.55	0.15	0.02	$-$ 0.11	$-$ 0.29	0.08
(22) LTSVR	0.34	11.96	0.48	0.53	0.28	0.19	0.04	0.34	0.62	18.55	0.75	0.28	0.08	0.02	$-$ 0.24	0.28
(23) Stepwise glm	0.18	6.37	0.26	0.69	0.47	0.00	$-$ 0.09	0.09	0.48	14.46	0.60	0.24	0.06	$-$ 0.21	$-$ 0.40	$-$ 0.02
(24) Neural networks	0.16	6.12	0.20	0.79	0.63	0.01	$-$ 0.06	0.08	0.65	20.61	0.79	0.35	0.13	0.19	$-$ 0.07	0.45

Best results (i.e. highest accuracy) are in bold.

*RMSE* root mean squared error, *R* Pearson correlation coefficient, (*R*$^2$) the coefficient of determination, *MDF* mean delta force, *LCI* lower confidence interval, *UPF* upper confidence interval, *GR* Gaussian regression, *K* Kernel.

**Table 4:** Summary of the performance of the algorithms for All Participants group, considering Input 3 as training data.

Function	1st Knee contact peak (N/body weight)								2nd Knee contact peak (N/body weight)							
	MAE	RPE	RMSE	R	R2	MDF	LCI	UCI	MAE	RPE	RMSE	R	R2	MDF	LCI	UCI
(1) Ensemble trees bagging)	0.27	9.56	0.34	0.80	0.64	0.20	0.11	0.29	0.42	11.77	0.58	0.11	0.01	$-$ 0.19	$-$ 0.38	0.00
(2) Ensemble trees (LSBoost)	0.26	9.16	0.36	0.76	0.58	$-$ 0.05	$-$ 0.17	0.08	0.54	15.55	0.74	0.00	0.00	$-$ 0.30	$-$ 0.54	$-$ 0.07
(3) Linear SVR	0.10	3.93	0.14	0.93	0.87	0.06	0.02	0.10	**0.16**	**4.71**	**0.21**	**0.94**	**0.88**	$-$ **0.07**	$-$ **0.14**	$-$ **0.01**
(4) Quadratic SVR	0.31	10.41	0.44	0.78	0.60	0.12	$-$ 0.03	0.27	0.20	5.61	0.26	0.92	0.84	$-$ 0.07	$-$ 0.16	0.01
(5) Cubic SVR	0.13	4.64	0.18	0.92	0.85	0.12	0.07	0.16	0.18	5.04	0.25	0.93	0.86	$-$ 0.07	$-$ 0.15	0.01
(6) Gaussian SVR	**0.09**	**3.37**	**0.12**	**0.94**	**0.88**	**0.01**	$-$ **0.03**	**0.05**	0.19	5.36	0.26	0.92	0.85	$-$ 0.07	$-$ 0.16	0.01
(7) Linear regression	0.67	21.98	0.99	0.79	0.63	0.61	0.35	0.88	0.37	10.23	0.51	0.46	0.21	$-$ 0.27	$-$ 0.42	$-$ 0.11
(8) Lasso regression	0.11	4.13	0.15	0.93	0.86	0.09	0.05	0.13	0.47	13.25	0.65	0.01	0.00	$-$ 0.31	$-$ 0.50	$-$ 0.11
(9) Ridge regression	0.15	5.38	0.20	0.92	0.85	0.14	0.10	0.19	0.32	9.32	0.44	0.61	0.37	$-$ 0.20	$-$ 0.33	$-$ 0.06
(10) Binary decision tree	0.21	7.40	0.25	0.83	0.69	0.05	$-$ 0.03	0.14	0.54	15.31	0.73	0.14	0.02	$-$ 0.29	$-$ 0.52	$-$ 0.06
(11) GR (K.-exponential)	0.11	4.22	0.15	0.92	0.84	0.06	0.01	0.10	0.38	10.47	0.55	0.23	0.05	$-$ 0.25	$-$ 0.42	$-$ 0.08
(12) GR (K.-squared exponential)	0.10	3.60	0.12	0.94	0.87	0.03	$-$ 0.01	0.07	0.23	6.57	0.32	0.87	0.76	$-$ 0.12	$-$ 0.22	$-$ 0.01
(13) GR (K.-matern 32)	0.09	3.46	0.11	0.94	0.88	0.02	$-$ 0.02	0.05	0.23	6.44	0.34	0.87	0.76	$-$ 0.15	$-$ 0.25	$-$ 0.04
(14) GR (K.-matern 52)	0.10	3.70	0.13	0.92	0.84	$-$ 0.01	$-$ 0.06	0.03	0.23	6.50	0.33	0.87	0.76	$-$ 0.13	$-$ 0.24	$-$ 0.03
(15) GR (K.-rational quadratic)	0.09	3.55	0.12	0.93	0.87	0.01	$-$ 0.04	0.05	0.23	6.57	0.32	0.87	0.76	$-$ 0.12	$-$ 0.22	$-$ 0.01
(16) ETSVR-Kernel Linear	0.13	4.75	0.17	0.86	0.74	0.03	$-$ 0.03	0.09	0.25	7.38	0.34	0.80	0.63	$-$ 0.14	$-$ 0.25	$-$ 0.04
(17) Kernel ridge regression	0.12	4.41	0.16	0.89	0.79	0.03	$-$ 0.02	0.09	0.29	8.29	0.38	0.72	0.52	$-$ 0.16	$-$ 0.28	$-$ 0.05
(18) Nyström ridge regression	0.13	4.70	0.16	0.88	0.78	0.01	$-$ 0.05	0.06	0.21	6.19	0.27	0.86	0.74	$-$ 0.05	$-$ 0.14	0.04
(19) DNNE	0.46	15.70	0.64	0.73	0.54	0.37	0.19	0.55	0.35	9.88	0.52	0.31	0.10	$-$ 0.18	$-$ 0.34	$-$ 0.01
(20) kNN weighted mean	0.39	13.83	0.47	0.71	0.50	0.26	0.13	0.40	0.55	16.21	0.76	0.15	0.02	$-$ 0.25	$-$ 0.50	0.00
(21) RKNNWTSVR	0.13	4.61	0.16	0.89	0.79	0.05	0.00	0.11	0.26	7.55	0.36	0.79	0.62	$-$ 0.18	$-$ 0.29	$-$ 0.07
(22) LTSVR	0.23	8.16	0.29	0.54	0.29	$-$ 0.09	$-$ 0.19	0.00	0.41	11.66	0.57	0.37	0.14	$-$ 0.28	$-$ 0.45	$-$ 0.11
(23) Stepwise glm	0.13	5.10	0.18	0.87	0.75	0.02	$-$ 0.04	0.09	0.45	13.31	0.58	0.13	0.02	$-$ 0.22	$-$ 0.40	$-$ 0.03
(24) Neural networks	0.13	4.56	0.16	0.90	0.80	$-$ 0.04	$-$ 0.10	0.01	0.20	6.35	0.23	0.92	0.85	$-$ 0.09	$-$ 0.16	$-$ 0.02

Best results (i.e. highest accuracy) are in bold.

*RMSE* root mean squared error, *R* Pearson correlation coefficient, (*R*$^2$) the coefficient of determination, *MDF* mean delta force, *LCI* lower confidence interval, *UPF* upper confidence interval, *GR* Gaussian regression, *K* Kernel.

Tables 5, 6, and 7 present the performance of each model for the OA group, considering Inputs 1, 2, and 3 as training data, respectively. When Input 1 was set up as training data, considering the 1st peak predictions, MAE ranged from 0.12 to 0.57. The highest accuracy was identified for Gaussian Regression (Kernel-matern 32) with a coefficient of determination of 0.86, but an excellent performance was also achieved by Gaussian Regression (Kernel-exponential) and Linear Regression. For the 2nd peak, the best accuracy was achieved by the DNNE regressor, with a coefficient of determination of 0.90 and an RPE lower than 5%.

Considering Input 2 as the training dataset, MAE ranged from 0.14 to 0.64 with the highest accuracy for 1st peak predictions identified for the Gaussian Regression (Kernel-matern 32) considering its highest coefficient of determination and an RPE lower than 7%. For the 2nd peak, the highest coefficient of determination was identified for the DNNE model, but with an MAE around 0.80.

MAE ranged from 0.07 to 1.11 for 1st peak predictions when Input 3 was used as the training dataset, being the Gaussian Regression (Kernel-matern 32) the model that presented the highest coefficient of determination. Excellent results were also identified for the Linear and Gaussian SVR, with an RPE lower than around 3%. For the 2nd peak predictions, MAE ranged from 0.15 to 0.73, with the highest accuracy coefficient of determination identified for the Linear SVR and the lowest RPE for the Neural Networks.

**Table 5:** Summary of the performance of the algorithms for the OA group, considering input 1 as training data.

Function	1st Knee contact peak (N/body weight)								2nd Knee contact peak (N/body weight)							
	MAE	RPE	RMSE	R	R2	MDF	LCI	UCI	MAE	RPE	RMSE	R	R2	MDF	LCI	UCI
(1) Ensemble trees (bagging)	0.28	9.43	0.36	0.47	0.22	$-$ 0.22	$-$ 0.36	$-$ 0.08	0.59	15.35	0.78	0.17	0.03	$-$ 0.58	$-$ 0.83	$-$ 0.33
(2) Ensemble trees (LSBoost)	0.46	15.61	0.52	0.27	0.08	$-$ 0.04	$-$ 0.29	0.20	0.50	14.20	0.73	0.58	0.34	$-$ 0.46	$-$ 0.73	$-$ 0.18
(3) Linear SVR	0.34	11.19	0.40	0.85	0.72	0.34	0.24	0.44	0.69	19.81	0.81	0.65	0.42	$-$ 0.67	$-$ 0.89	$-$ 0.46
(4) Quadratic SVR	0.57	19.08	0.66	0.62	0.39	$-$ 0.50	$-$ 0.71	$-$ 0.29	0.66	18.16	0.79	0.57	0.33	$-$ 0.66	$-$ 0.87	$-$ 0.45
(5) Cubic SVR	0.42	13.93	0.48	0.59	0.35	$-$ 0.37	$-$ 0.52	$-$ 0.22	1.27	41.48	1.94	0.74	0.55	$-$ 1.23	$-$ 1.95	$-$ 0.50
(6) Gaussian SVR	0.18	6.13	0.21	0.85	0.72	$-$ 0.16	$-$ 0.22	$-$ 0.09	0.52	14.79	0.58	0.14	0.02	$-$ 0.26	$-$ 0.51	$-$ 0.01
(7) Linear regression	0.17	5.79	0.21	0.84	0.70	0.13	0.05	0.21	0.75	20.26	0.91	0.18	0.03	$-$ 0.70	$-$ 0.98	$-$ 0.42
(8) Lasso regression	0.17	5.63	0.24	0.68	0.47	0.11	0.01	0.21	0.71	19.57	0.84	0.45	0.20	$-$ 0.68	$-$ 0.92	$-$ 0.44
(9) Ridge regression	0.28	9.61	0.41	0.54	0.29	0.25	0.10	0.41	0.63	17.56	0.75	0.62	0.39	$-$ 0.61	$-$ 0.82	$-$ 0.40
(10) Binary decision tree	0.38	13.00	0.45	0.71	0.51	$-$ 0.37	$-$ 0.49	$-$ 0.24	0.52	13.82	0.65	0.09	0.01	$-$ 0.33	$-$ 0.60	$-$ 0.06
(11) GR (K.-exponential)	0.15	5.11	0.18	0.84	0.70	$-$ 0.10	$-$ 0.17	$-$ 0.02	0.51	13.57	0.63	0.28	0.08	$-$ 0.38	$-$ 0.62	$-$ 0.14
(12) GR (K.-squared exponential)	0.26	8.57	0.28	0.85	0.72	$-$ 0.24	$-$ 0.31	$-$ 0.18	0.52	14.52	0.59	0.08	0.01	$-$ 0.26	$-$ 0.51	0.00
(13) GR (K.-matern 32)	**0.12**	**4.13**	**0.14**	**0.92**	**0.86**	$-$ **0.10**	$-$ **0.15**	$-$ **0.05**	0.53	14.80	0.61	0.02	0.00	$-$ 0.30	$-$ 0.56	$-$ 0.04
(14) GR (K.-matern 52)	0.22	7.40	0.24	0.87	0.76	$-$ 0.21	$-$ 0.27	$-$ 0.15	0.53	14.89	0.61	0.02	0.00	$-$ 0.28	$-$ 0.54	$-$ 0.02
(15) GR (K.-rational quadratic)	0.21	7.18	0.23	0.87	0.76	$-$ 0.20	$-$ 0.26	$-$ 0.14	0.53	14.79	0.60	0.01	0.00	$-$ 0.27	$-$ 0.53	$-$ 0.02
(16) ETSVR-Kernel Linear	0.40	13.63	0.56	0.53	0.28	0.39	0.20	0.59	0.67	18.59	0.80	0.59	0.35	$-$ 0.67	$-$ 0.88	$-$ 0.45
(17) Kernel ridge regression	0.44	14.88	0.59	0.55	0.30	0.43	0.24	0.63	0.66	18.46	0.79	0.56	0.32	$-$ 0.64	$-$ 0.86	$-$ 0.41
(18) Nyström ridge regression	0.53	17.66	0.71	0.49	0.24	0.51	0.28	0.75	0.35	9.29	0.55	0.51	0.26	$-$ 0.26	$-$ 0.49	$-$ 0.03
(19) DNNE	0.35	11.61	0.43	0.74	0.55	0.28	0.12	0.43	**0.17**	**4.73**	**0.22**	**0.95**	**0.90**	$-$ **0.15**	$-$ **0.23**	$-$ **0.07**
(20) kNN weighted mean	0.32	10.38	0.37	0.65	0.43	$-$ 0.31	$-$ 0.40	$-$ 0.23	0.50	13.21	0.66	0.14	0.02	$-$ 0.39	$-$ 0.65	$-$ 0.14
(21) RKNNWTSVR	0.36	12.14	0.49	0.61	0.38	0.35	0.19	0.51	0.71	19.82	0.83	0.54	0.30	$-$ 0.69	$-$ 0.92	$-$ 0.46
(22) LTSVR	0.44	14.79	0.64	0.43	0.18	0.42	0.19	0.65	0.56	15.78	0.69	0.66	0.43	$-$ 0.55	$-$ 0.75	$-$ 0.35
(23) Stepwise glm	0.17	5.60	0.23	0.71	0.50	$-$ 0.16	$-$ 0.24	$-$ 0.08	0.61	17.14	0.73	0.67	0.45	$-$ 0.61	$-$ 0.80	$-$ 0.41
(24) Neural networks	0.24	8.00	0.28	0.77	0.59	0.06	$-$ 0.07	0.19	0.79	20.56	1.02	0.26	0.07	$-$ 0.77	$-$ 1.10	$-$ 0.45

Best results (i.e. highest accuracy) are in bold.

*RMSE* root mean squared error, *R* Pearson correlation coefficient, (*R*$^2$) the coefficient of determination, *MDF* mean delta force, *LCI* lower confidence interval, *UPF* upper confidence interval, *GR* Gaussian regression, *K* Kernel.

**Table 6:** Summary of the performance of the algorithms for the OA group, considering Input 2 as training data.

Function	1st Knee contact peak (N/body weight)								2nd Knee contact peak (N/body weight)							
	MAE	RPE	RMSE	R	R2	MDF	LCI	UCI	MAE	RPE	RMSE	R	R2	MDF	LCI	UCI
(1) Ensemble trees (bagging)	0.31	10.28	0.39	0.36	0.13	0.03	$-$ 0.16	0.21	0.58	14.80	0.77	0.15	0.02	$-$ 0.57	$-$ 0.82	$-$ 0.31
(2) Ensemble trees (LSBoost)	0.38	12.65	0.47	0.12	0.01	$-$ 0.07	$-$ 0.29	0.16	0.72	20.92	0.88	0.62	0.39	$-$ 0.69	$-$ 0.95	$-$ 0.42
(3) Linear SVR	0.34	11.42	0.41	0.81	0.66	0.34	0.23	0.45	0.40	10.54	0.54	0.66	0.43	$-$ 0.36	$-$ 0.55	$-$ 0.17
(4) Quadratic SVR	0.38	12.41	0.46	0.75	0.57	$-$ 0.16	$-$ 0.37	0.04	0.41	13.55	0.51	0.39	0.15	$-$ 0.39	$-$ 0.55	$-$ 0.23
(5) Cubic SVR	0.26	8.23	0.33	0.34	0.12	$-$ 0.22	$-$ 0.34	$-$ 0.10	0.72	19.68	0.86	0.49	0.24	$-$ 0.71	$-$ 0.94	$-$ 0.48
(6) Gaussian SVR	0.19	6.46	0.21	0.86	0.74	$-$ 0.15	$-$ 0.22	$-$ 0.09	0.60	17.70	0.69	0.18	0.03	$-$ 0.15	$-$ 0.47	0.18
(7) Linear regression	0.17	5.77	0.20	0.83	0.69	0.12	0.04	0.20	0.64	16.88	0.81	0.10	0.01	$-$ 0.58	$-$ 0.85	$-$ 0.31
(8) Lasso regression	0.20	6.70	0.26	0.49	0.24	0.03	$-$ 0.10	0.15	0.77	20.48	0.98	0.01	0.00	$-$ 0.76	$-$ 1.06	$-$ 0.46
(9) Ridge regression	0.33	11.10	0.44	0.27	0.08	0.17	$-$ 0.02	0.37	0.68	18.64	0.86	0.30	0.09	$-$ 0.41	$-$ 0.77	$-$ 0.04
(10) Binary decision tree	0.64	21.29	0.75	0.10	0.01	0.14	$-$ 0.22	0.49	0.63	16.44	0.78	0.05	0.00	$-$ 0.52	$-$ 0.80	$-$ 0.25
(11) GR (K.-exponential)	0.26	8.57	0.28	0.85	0.72	-0.25	$-$ 0.31	$-$ 0.18	0.62	17.65	0.66	0.02	0.00	$-$ 0.30	$-$ 0.58	$-$ 0.02
(12) GR (K.-squared exponential)	0.19	6.01	0.25	0.78	0.61	$-$ 0.19	$-$ 0.27	$-$ 0.10	0.53	14.93	0.63	0.27	0.07	$-$ 0.22	$-$ 0.50	0.07
(13) GR (K.-matern 32)	**0.20**	**6.78**	**0.22**	**0.88**	**0.77**	$-$ **0.18**	$-$ **0.24**	$-$ **0.12**	0.61	16.64	0.72	0.31	0.10	$-$ 0.41	$-$ 0.69	$-$ 0.12
(14) GR (K.-matern 52)	0.22	7.40	0.24	0.87	0.76	$-$ 0.21	$-$ 0.27	$-$ 0.15	0.63	17.69	0.69	0.14	0.02	$-$ 0.37	$-$ 0.65	$-$ 0.08
(15) GR (K.$-$ rational quadratic)	0.21	7.18	0.23	0.87	0.76	$-$ 0.20	$-$ 0.26	$-$ 0.14	0.62	17.66	0.66	0.04	0.00	$-$ 0.32	$-$ 0.60	$-$ 0.04
(16) ETSVR-Kernel linear	0.39	13.27	0.54	0.30	0.09	0.28	0.06	0.50	0.53	14.58	0.68	0.08	0.01	$-$ 0.29	$-$ 0.58	0.01
(17) Kernel ridge regression	0.52	17.44	0.69	0.21	0.04	0.37	0.09	0.65	0.55	15.16	0.72	0.01	0.00	$-$ 0.33	$-$ 0.63	$-$ 0.02
(18) Nyström ridge regression	0.57	18.96	0.78	0.01	0.00	0.25	$-$ 0.10	0.61	0.99	28.40	1.22	0.56	0.31	$-$ 0.38	$-$ 0.94	0.18
(19) DNNE	0.41	13.75	0.53	0.65	0.42	0.34	0.15	0.54	**0.81**	**24.72**	**0.93**	**0.79**	**0.62**	$-$ **0.74**	$-$ **1.01**	$-$ **0.47**
(20) kNN weighted mean	0.32	10.38	0.37	0.65	0.43	$-$ 0.31	$-$ 0.40	$-$ 0.23	0.50	13.21	0.66	0.14	0.02	$-$ 0.39	$-$ 0.65	$-$ 0.14
(21) RKNNWTSVR	0.32	10.94	0.46	0.57	0.32	0.31	0.14	0.47	0.48	12.55	0.65	0.51	0.26	$-$ 0.46	$-$ 0.68	$-$ 0.25
(22) LTSVR	0.47	15.59	0.61	0.17	0.03	0.28	0.02	0.54	0.73	20.57	0.90	0.38	0.14	$-$ 0.34	$-$ 0.74	0.06
(23) Stepwise glm	0.19	6.37	0.29	0.48	0.23	$-$ 0.02	$-$ 0.16	0.12	0.61	17.14	0.73	0.67	0.45	$-$ 0.61	$-$ 0.80	$-$ 0.41
(24) Neural networks	0.14	4.62	0.17	0.80	0.64	$-$ 0.05	$-$ 0.12	0.03	0.80	24.08	0.96	0.56	0.31	$-$ 0.03	$-$ 0.49	0.43

Best results (i.e. highest accuracy) are in bold.

*RMSE* root mean squared error, *R* Pearson correlation coefficient, (*R*$^2$) the coefficient of determination, *MDF* mean delta force, *LCI* lower confidence interval, *UPF* upper confidence interval, *GR* Gaussian regression, *K* Kernel.

**Table 7:** Summary of the performance of the algorithms for the OA group, considering Input 3 as training data.

Function	1st Knee contact peak (N/body weight)								2nd Knee contact peak (N/body weight)							
	MAE	RPE	RMSE	R	R2	MDF	LCI	UCI	MAE	RPE	RMSE	R	R2	MDF	LCI	UCI
(1) Ensemble trees (bagging)	0.35	11.99	0.42	0.69	0.48	0.30	0.16	0.44	0.55	14.07	0.73	0.13	0.02	$-$ 0.51	$-$0.76	$-$0.26
(2) Ensemble trees (LSBoost)	0.25	8.55	0.37	0.44	0.20	$-$0.02	$-$0.19	0.16	0.73	19.95	0.91	0.02	0.00	$-$0.65	$-$0.96	$-$0.34
(3) Linear SVR	0.07	2.48	0.11	0.91	0.82	$-$0.01	$-$0.06	0.04	**0.21**	**5.80**	**0.26**	**0.96**	**0.92**	$-$ **0.20**	$-$ **0.28**	$-$ **0.13**
(4) Quadratic SVR	0.48	15.64	0.59	0.81	0.66	0.16	$-$0.12	0.43	0.27	7.42	0.34	0.93	0.87	$-$0.18	$-$0.32	$-$0.05
(5) Cubic SVR	0.15	5.10	0.21	0.86	0.74	0.14	0.07	0.21	0.25	6.66	0.32	0.94	0.88	$-$0.18	$-$0.30	$-$0.05
(6) Gaussian SVR	0.09	3.16	0.11	0.92	0.85	$-$0.03	$-$0.09	0.02	0.27	7.26	0.33	0.93	0.86	$-$0.18	$-$0.31	$-$0.04
(7) Linear regression	1.11	36.04	1.32	0.84	0.70	1.10	0.75	1.45	0.53	13.86	0.67	0.44	0.19	$-$0.47	$-$0.70	$-$0.25
(8) Lasso regression	0.10	3.51	0.16	0.84	0.71	0.07	0.00	0.14	0.68	18.10	0.84	0.34	0.12	$-$0.67	$-$0.92	$-$0.43
(9) Ridge regression	0.17	5.80	0.23	0.89	0.79	0.17	0.10	0.24	0.47	12.80	0.57	0.77	0.60	$-$0.47	$-$0.63	$-$0.31
(10) Binary decision tree	0.17	5.77	0.22	0.72	0.51	0.02	$-$0.08	0.13	0.75	19.96	0.92	0.09	0.01	$-$0.69	$-$0.98	$-$0.39
(11) GR (K.-exponential)	0.12	4.16	0.15	0.89	0.79	0.06	$-$0.01	0.12	0.54	14.12	0.71	0.53	0.28	$-$0.54	$-$0.76	$-$0.32
(12) GR (K.-squared exponential)	0.10	3.43	0.12	0.93	0.86	0.00	$-$0.05	0.06	0.34	9.17	0.42	0.92	0.84	$-$0.25	$-$0.42	$-$0.09
(13) GR (K.-matern 32)	**0.10**	**3.34**	**0.11**	**0.93**	**0.87**	$-$ **0.02**	$-$ **0.07**	**0.04**	0.34	8.99	0.44	0.92	0.85	$-$0.31	$-$0.46	$-$0.15
(14) GR (K.-matern 52)	0.11	3.88	0.15	0.92	0.84	$-$0.07	$-$0.13	$-$0.01	0.34	9.12	0.43	0.92	0.85	$-$0.28	$-$0.44	$-$0.13
(15) GR (K.-rational quadratic)	0.10	3.43	0.12	0.93	0.86	$-$0.04	$-$0.09	0.02	0.34	9.17	0.42	0.92	0.84	$-$0.25	$-$0.42	$-$0.09
(16) ETSVR-Kernel linear	0.11	3.53	0.16	0.85	0.72	$-$0.06	$-$0.13	0.01	0.36	9.84	0.43	0.89	0.79	$-$0.35	$-$0.47	$-$0.23
(17) Kernel ridge regression	0.10	3.19	0.14	0.88	0.77	$-$0.05	$-$0.11	0.01	0.41	11.15	0.50	0.85	0.71	$-$0.40	$-$0.54	$-$0.27
(18) Nyström ridge regression	0.24	7.80	0.31	0.53	0.28	0.00	$-$0.15	0.14	0.61	17.07	0.77	0.60	0.36	$-$0.59	$-$0.83	$-$0.36
(19) DNNE	0.69	22.91	0.82	0.67	0.45	0.68	0.46	0.90	0.57	15.35	0.69	0.04	0.00	$-$0.32	$-$0.61	$-$0.03
(20) kNN weighted mean	0.31	10.50	0.41	0.73	0.53	0.30	0.16	0.43	0.59	15.33	0.78	0.03	0.00	$-$0.54	$-$0.81	$-$0.27
(21) RKNNWTSVR	0.11	3.50	0.15	0.87	0.76	$-$0.01	$-$0.09	0.06	0.39	10.73	0.47	0.86	0.75	$-$0.38	$-$0.51	$-$0.25
(22) LTSVR	0.28	8.89	0.34	0.49	0.24	$-$0.27	$-$0.37	$-$0.17	0.62	16.98	0.75	0.61	0.37	$-$0.62	$-$0.82	$-$0.42
(23) Stepwise glm	0.12	3.84	0.15	0.84	0.70	$-$0.08	$-$0.14	$-$0.01	0.58	15.47	0.71	0.65	0.42	$-$0.58	$-$0.77	$-$0.38
(24) Neural networks	0.15	5.06	0.19	0.88	0.77	$-$0.07	$-$0.15	0.01	0.15	4.23	0.17	0.95	0.91	$-$0.06	$-$0.14	0.01

Best results (i.e. highest accuracy) are in bold.

*RMSE* root mean squared error, *R* Pearson correlation coefficient, (*R*$^2$) the coefficient of determination, *MDF* mean delta force, *LCI* lower confidence interval, *UPF* upper confidence interval, *GR* Gaussian regression, *K* Kernel.

Finally, independent tests were also performed for healthy participants, labelled as the Control group. Tables 8, 9, and 10 present algorithms performance taking into consideration Input 1, 2, and 3 as the training datasets, respectively. Input 1 as the training dataset resulted in MAE ranging from 0.17 and 0.50 for the 1st peak prediction, with the highest coefficient of determination achieved by Gaussian regressors (Kernel matern 52 and rational quadratic). Excellent performance was also identified for the Kernel squared exponential Gaussian model, with an RPE lower than 7%. For the 2nd peak prediction, MAE ranged from 0.38 to 0.72, with the highest accuracy achieved by Gaussian Regressor (Kernel squared exponential).

When Input 2 was set as training data, for the 1st peak prediction, MAE ranged from 0.17 to 0.47. The highest accuracy was identified for the Cubic SVR, with an RPE lower than 8% and a coefficient of determination higher than 0.70. The Gaussian regressors (Kernel matern 32 and 52) also achieved promising performance, with an RPE lower than 7%. For the 2nd peak prediction, Quadratic SVR achieved the highest performance, with an RPE lower than 6% and a coefficient of determination of 0.80. Considering all models, MAE ranged from 0.14 to 0.55.

MAE ranged from 0.10 to 0.26 for the 1st peak prediction, considering Input 3 as the training dataset. The lowest RPE was identified for the Cubic SVR model and the highest coefficient of determination (0.98) for the Lasso Regression. For the 2nd peak, MAE ranged from 0.09 to 0.30. The lowest RPE was identified for the Gaussian SVR, while Kernel Ridge Regression presented the highest coefficient of determination (0.92).

**Table 8:** Summary of the algorithms’ performance for the Control group, considering input 1 as training data.

Function	1st Knee contact peak (N/body weight)								2nd Knee contact peak (N/body weight)							
	MAE	RPE	RMSE	R	R2	MDF	LCI	UCI	MAE	RPE	RMSE	R	R2	MDF	LCI	UCI
(1) Ensemble trees (bagging)	0.21	7.73	0.25	0.72	0.52	$-$ 0.06	$-$ 0.20	0.07	0.30	10.37	0.34	0.25	0.06	0.18	0.02	0.34
(2) Ensemble trees (LSBoost)	0.23	8.81	0.26	0.68	0.46	0.02	$-$ 0.13	0.16	0.40	13.95	0.48	0.53	0.28	0.37	0.21	0.54
(3) Linear SVR	0.22	9.23	0.28	0.58	0.34	0.09	$-$ 0.06	0.23	0.43	15.05	0.49	0.54	0.29	0.43	0.30	0.56
(4) Quadratic SVR	0.17	6.73	0.20	0.80	0.65	0.01	$-$ 0.10	0.12	0.50	17.37	0.54	0.62	0.39	0.50	0.39	0.61
(5) Cubic SVR	0.20	7.78	0.25	0.70	0.49	$-$ 0.07	$-$ 0.20	0.05	0.46	15.84	0.50	0.66	0.44	0.46	0.35	0.57
(6) Gaussian SVR	0.20	7.62	0.25	0.83	0.69	$-$ 0.16	$-$ 0.26	$-$ 0.06	0.42	14.55	0.45	0.78	0.61	0.42	0.33	0.51
(7) Linear regression	0.23	9.47	0.30	0.57	0.33	0.13	$-$ 0.02	0.27	0.39	14.02	0.47	0.22	0.05	0.39	0.25	0.54
(8) Lasso regression	0.25	10.38	0.30	0.51	0.26	0.09	$-$ 0.07	0.25	0.41	14.37	0.46	0.47	0.22	0.41	0.28	0.53
(9) Ridge regression	0.28	11.82	0.36	0.29	0.09	0.12	$-$ 0.06	0.31	0.56	19.46	0.60	0.51	0.26	0.56	0.43	0.68
(10) Binary decision tree	0.23	8.63	0.29	0.72	0.52	$-$ 0.08	$-$ 0.23	0.08	0.43	14.78	0.48	0.20	0.04	0.22	$-$ 0.01	0.46
(11) GR (K.-exponential)	0.19	6.98	0.24	0.84	0.70	-0.16	$-$ 0.26	$-$ 0.06	0.42	14.76	0.46	0.74	0.55	0.42	0.33	0.52
(12) GR (K.-squared exponential)	0.17	6.56	0.21	0.83	0.69	$-$ 0.12	$-$ 0.22	$-$ 0.02	**0.38**	**13.28**	**0.41**	**0.78**	**0.61**	**0.38**	**0.29**	**0.47**
(13) GR (K.-matern 32)	0.23	9.65	0.29	0.57	0.32	0.10	$-$ 0.05	0.25	0.40	13.93	0.43	0.78	0.60	0.40	0.31	0.49
(14) GR (K.-matern 52)	**0.18**	**6.63**	**0.22**	**0.84**	**0.71**	$-$ **0.13**	$-$ **0.23**	$-$ **0.04**	0.39	13.71	0.43	0.78	0.60	0.39	0.30	0.48
(15) GR (K.-rational quadratic)	**0.18**	**6.65**	**0.22**	**0.84**	**0.71**	$-$ **0.13**	$-$ **0.23**	$-$ **0.03**	0.38	13.40	0.42	0.78	0.61	0.38	0.30	0.47
(16) ETSVR-Kernel linear	0.28	11.36	0.33	0.45	0.20	0.09	$-$ 0.08	0.26	0.46	16.11	0.51	0.48	0.23	0.46	0.33	0.59
(17) Kernel ridge regression	0.27	11.01	0.32	0.48	0.23	0.08	$-$ 0.09	0.25	0.57	19.75	0.61	0.47	0.23	0.57	0.44	0.69
(18) Nyström ridge regression	0.29	11.66	0.34	0.44	0.19	0.08	$-$ 0.10	0.26	0.58	20.34	0.63	0.49	0.24	0.58	0.46	0.71
(19) DNNE	0.50	18.28	0.55	0.65	0.42	$-$ 0.50	$-$ 0.63	$-$ 0.36	0.42	14.44	0.52	0.05	0.00	0.26	0.01	0.50
(20) kNN weighted Mean	0.39	14.15	0.44	0.66	0.44	$-$ 0.36	$-$ 0.50	$-$ 0.22	0.25	8.96	0.30	0.21	0.04	0.15	0.01	0.30
(21) RKNNWTSVR	0.26	10.44	0.30	0.50	0.25	0.08	$-$ 0.09	0.24	0.40	14.03	0.46	0.41	0.17	0.40	0.26	0.53
(22) LTSVR	0.30	12.43	0.36	0.33	0.11	0.11	$-$ 0.08	0.30	0.56	19.55	0.61	0.54	0.29	0.56	0.44	0.69
(23) Stepwise glm	0.25	10.02	0.28	0.59	0.35	0.06	$-$ 0.10	0.21	0.31	11.12	0.37	0.38	0.14	0.28	0.15	0.41
(24) Neural networks	0.27	10.78	0.32	0.36	0.13	$-$ 0.01	$-$ 0.19	0.16	0.72	24.81	0.78	0.39	0.15	0.72	0.55	0.88

Best results (i.e. highest accuracy) are in bold.

*RMSE* root mean squared error, *R* Pearson correlation coefficient, (*R*$^2$) the coefficient of determination, *MDF* mean delta force, *LCI* lower confidence interval, *UPF* upper confidence interval, *GR* Gaussian regression, *K* Kernel.

**Table 9:** Summary of the performance of the algorithms for the control group, considering Input 2 as training data.

Function	1st Knee contact peak (N/body weight)								2nd Knee contact peak (N/body weight)							
	MAE	RPE	RMSE	R	R2	MDF	LCI	UCI	MAE	RPE	RMSE	R	R2	MDF	LCI	UCI
(1) Ensemble trees (bagging)	0.23	8.72	0.29	0.61	0.37	0.03	$-$ 0.13	0.19	0.29	9.96	0.32	0.33	0.11	0.16	0.01	0.31
(2) Ensemble trees (LSBoost)	0.26	9.59	0.31	0.59	0.35	$-$ 0.03	$-$ 0.20	0.14	0.48	16.99	0.58	0.25	0.06	0.48	0.31	0.66
(3) Linear SVR	0.21	8.87	0.27	0.63	0.39	0.08	$-$ 0.06	0.22	0.34	11.82	0.38	0.76	0.58	0.34	0.24	0.43
(4) Quadratic SVR	0.19	7.37	0.21	0.81	0.65	0.08	$-$ 0.03	0.19	**0.14**	**5.67**	**0.17**	**0.89**	**0.80**	**0.04**	$-$ **0.05**	**0.13**
(5) Cubic SVR	**0.17**	**7.26**	**0.22**	**0.86**	**0.74**	**0.14**	**0.04**	**0.24**	0.28	9.72	0.31	0.83	0.69	0.27	0.19	0.35
(6) Gaussian SVR	0.19	7.35	0.24	0.81	0.66	$-$ 0.14	$-$ 0.24	$-$ 0.04	0.36	12.26	0.39	0.86	0.74	0.36	0.28	0.44
(7) Linear regression	0.23	9.38	0.30	0.58	0.34	0.13	$-$ 0.02	0.28	0.38	13.53	0.45	0.31	0.10	0.38	0.24	0.52
(8) Lasso regression	0.19	7.57	0.22	0.80	0.64	0.10	$-$ 0.01	0.21	0.43	14.86	0.46	0.79	0.62	0.43	0.34	0.52
(9) Ridge regression	0.20	8.08	0.24	0.77	0.59	0.11	0.00	0.23	0.47	15.95	0.48	0.89	0.79	0.47	0.40	0.53
(10) Binary decision tree	0.24	9.02	0.34	0.66	0.43	0.07	$-$ 0.11	0.25	0.46	16.27	0.52	0.44	0.19	0.46	0.33	0.60
(11) GR (K.-exponential)	0.17	6.56	0.21	0.83	0.69	$-$ 0.12	$-$ 0.22	$-$ 0.02	0.28	9.61	0.31	0.78	0.60	0.27	0.18	0.35
(12) GR (K.-squared exponential)	0.20	7.70	0.21	0.83	0.69	$-$ 0.04	$-$ 0.15	0.08	0.29	10.01	0.33	0.75	0.56	0.28	0.19	0.37
(13) GR (K.-matern 32)	0.17	6.47	0.22	0.85	0.71	$-$ 0.13	$-$ 0.23	$-$ 0.04	0.33	11.37	0.36	0.76	0.58	0.32	0.23	0.42
(14) GR (K.-matern 52)	0.18	6.63	0.22	0.84	0.71	$-$ 0.13	$-$ 0.23	$-$ 0.04	0.29	10.21	0.33	0.74	0.54	0.28	0.19	0.38
(15) GR (K.-rational quadratic)	0.18	6.65	0.22	0.84	0.71	$-$ 0.13	$-$ 0.23	$-$ 0.03	0.28	9.70	0.31	0.77	0.59	0.27	0.18	0.36
(16) ETSVR-Kernel linear	0.22	8.81	0.26	0.70	0.48	0.11	$-$ 0.02	0.24	0.39	13.58	0.41	0.90	0.80	0.39	0.33	0.46
(17) Kernel ridge regression	0.19	7.35	0.21	0.79	0.62	0.07	$-$ 0.05	0.18	0.46	15.73	0.47	0.91	0.82	0.46	0.40	0.52
(18) Nyström ridge regression	0.21	8.29	0.24	0.71	0.50	0.06	$-$ 0.07	0.19	0.47	15.73	0.51	0.83	0.69	0.46	0.34	0.58
(19) DNNE	0.47	17.33	0.52	0.78	0.60	$-$ 0.47	$-$ 0.59	$-$ 0.35	0.55	18.80	0.75	0.22	0.05	0.21	$-$ 0.19	0.60
(20) kNN weighted mean	0.39	14.15	0.44	0.66	0.44	$-$ 0.36	$-$ 0.50	$-$ 0.22	0.25	8.96	0.30	0.21	0.04	0.15	0.01	0.30
(21) RKNNWTSVR	0.23	9.46	0.28	0.56	0.32	0.07	$-$ 0.08	0.22	0.34	12.03	0.38	0.75	0.56	0.34	0.24	0.44
(22) LTSVR	0.19	7.43	0.21	0.79	0.62	0.08	$-$ 0.03	0.19	0.47	16.02	0.49	0.88	0.78	0.47	0.40	0.55
(23) Stepwise glm	0.16	6.36	0.20	0.79	0.63	0.02	$-$ 0.09	0.13	0.31	11.12	0.37	0.38	0.14	0.28	0.15	0.41
(24) Neural networks	0.19	8.00	0.24	0.75	0.57	0.08	$-$ 0.04	0.20	0.47	16.26	0.50	0.69	0.48	0.47	0.36	0.57

Best results (i.e. highest accuracy) are in bold.

*RMSE* root mean squared error, *R* Pearson correlation coefficient, (*R*$^2$) the coefficient of determination, *MDF* mean delta force, *LCI* lower confidence interval, *UPF* upper confidence interval, *GR* Gaussian regression, *K* Kernel.

**Table 10:** Summary of the performance of the algorithms for the control group, considering Input 3 as training data.

Function	1st Knee contact peak (N/body weight)								2nd Knee contact peak (N/body weight)							
	MAE	RPE	RMSE	R	R2	MDF	LCI	UCI	MAE	RPE	RMSE	R	R2	MDF	LCI	UCI
(1) Ensemble trees (bagging)	0.17	6.52	0.20	0.82	0.68	0.08	$-$ 0.03	0.18	0.25	8.89	0.31	0.54	0.29	0.21	0.10	0.33
(2) Ensemble trees (LSBoost)	0.26	9.93	0.34	0.88	0.77	$-$ 0.08	$-$ 0.26	0.10	0.29	10.05	0.43	0.54	0.29	0.13	$-$ 0.10	0.36
(3) Linear SVR	0.14	5.75	0.17	0.96	0.93	0.14	0.09	0.19	0.10	3.34	0.12	0.95	0.90	0.09	0.04	0.13
(4) Quadratic SVR	0.10	3.88	0.12	0.96	0.92	0.08	0.02	0.13	0.10	3.35	0.11	0.94	0.88	0.06	0.01	0.12
(5) Cubic SVR	**0.10**	**4.07**	**0.13**	**0.97**	**0.95**	**0.09**	**0.03**	**0.14**	0.09	3.02	0.10	0.95	0.91	0.07	0.02	0.11
(6) Gaussian SVR	0.08	3.63	0.12	0.97	0.94	0.07	0.01	0.12	**0.09**	**2.99**	**0.10**	**0.95**	**0.90**	**0.06**	**0.02**	**0.11**
(7) Linear regression	0.12	4.40	0.14	0.90	0.81	0.01	$-$ 0.07	0.08	0.17	5.69	0.20	0.61	0.38	$-$ 0.01	$-$ 0.12	0.10
(8) Lasso regression	**0.12**	**4.92**	**0.15**	**0.99**	**0.98**	**0.11**	**0.06**	**0.17**	0.20	7.19	0.27	0.56	0.31	0.16	0.04	0.27
(9) Ridge regression	0.12	4.86	0.15	0.96	0.93	0.11	0.05	0.16	0.14	4.96	0.16	0.95	0.90	0.14	0.09	0.18
(10) Binary decision tree	0.25	9.44	0.29	0.89	0.79	0.09	$-$ 0.06	0.24	0.27	9.49	0.37	0.37	0.14	0.21	0.04	0.38
(11) GR (K.-exponential)	0.10	4.29	0.14	0.96	0.92	0.06	$-$ 0.01	0.13	0.17	5.92	0.23	0.60	0.37	0.11	$-$ 0.01	0.22
(12) GR (K.-squared exponential)	0.09	3.80	0.12	0.96	0.92	0.06	0.00	0.12	0.10	3.32	0.12	0.92	0.85	0.06	0.00	0.11
(13) GR (K.-matern 32)	0.08	3.62	0.11	0.97	0.93	0.06	0.00	0.11	0.10	3.25	0.12	0.92	0.84	0.06	0.00	0.11
(14) GR (K.-matern 52)	0.08	3.47	0.11	0.97	0.94	0.06	0.01	0.11	0.10	3.22	0.11	0.92	0.85	0.06	0.00	0.11
(15) GR (K.-rational quadratic)	0.09	3.69	0.12	0.96	0.92	0.06	0.00	0.12	0.10	3.32	0.12	0.92	0.85	0.06	0.00	0.11
(16) ETSVR-Kernel linear	0.16	6.28	0.19	0.94	0.89	0.15	0.09	0.21	0.12	4.29	0.15	0.94	0.88	0.12	0.07	0.17
(17) Kernel ridge regression	0.15	5.93	0.18	0.94	0.89	0.14	0.08	0.20	**0.14**	**4.72**	**0.16**	**0.96**	**0.92**	**0.14**	**0.10**	**0.18**
(18) Nyström ridge regression	0.17	7.04	0.21	0.93	0.87	0.17	0.10	0.23	0.22	7.67	0.28	0.36	0.13	0.05	$-$ 0.10	0.21
(19) DNNE	0.18	6.68	0.25	0.69	0.47	$-$ 0.02	$-$ 0.16	0.11	0.09	3.04	0.10	0.92	0.85	0.00	$-$ 0.05	0.06
(20) kNN weighted mean	0.20	7.83	0.24	0.67	0.45	$-$ 0.02	$-$ 0.15	0.12	0.28	10.37	0.40	0.18	0.03	0.25	0.08	0.42
(21) RKNNWTSVR	0.15	6.00	0.18	0.94	0.89	0.14	0.08	0.20	0.10	3.59	0.13	0.92	0.85	0.08	0.02	0.14
(22) LTSVR	0.18	7.25	0.22	0.84	0.71	0.13	0.03	0.22	0.15	5.02	0.17	0.93	0.87	0.14	0.09	0.19
(23) Stepwise glm	0.16	6.68	0.20	0.98	0.96	0.15	0.08	0.23	0.30	10.62	0.35	0.33	0.11	0.23	0.09	0.38
(24) Neural networks	0.10	3.94	0.12	0.94	0.89	$-$ 0.01	$-$ 0.07	0.06	0.26	9.00	0.28	0.66	0.43	$-$ 0.12	$-$ 0.26	0.01

Best results (i.e. highest accuracy) are in bold.

*RMSE* root mean squared error, *R* Pearson correlation coefficient, (*R*$^2$) the coefficient of determination, *MDF* mean delta force, *LCI* lower confidence interval, *UPF* upper confidence interval, *GR* Gaussian regression, *K* Kernel.

## Discussion

This study presented a comprehensive evaluation of different machine learning models to predict tibiofemoral contact forces during the gait task of healthy and knee OA patients. Results were analyzed in light of different training datasets. The main results were: (a) accurate predictions of the tibiofemoral contact forces were possible using machine learning algorithms, independent of the participants’ features (healthy or OA); (b) in general, the 1st force peak was not very sensitive to changes in the input dataset, reaching promising results only with kinetic and kinematic primary data; (c) in general, the 2nd force peak was sensitive to changes in the input data, once better results were achieved when a greater range of variables was defined as training data; (d) when analyzed independently by the pre-trained machine learning models, the OA and Control groups presented promising accuracy to predict both peaks with primary data while using lower limbs joint moments information.

Machine learning algorithms’ performance was evaluated considering a different number of predicting variables (labelled as Input 1, 2, and 3) as the training dataset. It is important to emphasize that the training dataset was composed of healthy and knee OA patients. Still, independent tests were performed considering a mixed group (labelled as All participants, with healthy and symptomatic individuals), and separated groups. No participants included in the training dataset were evaluated during the independent tests, assuring that the model generalizes well to new unseen data and does not overfit due to dependency between training and test split data^38^. In general, our results presented similar or higher accuracy for knee contact forces prediction when compared to a previous study with total knee replacement patients^13^ that reported mean correlation coefficients ranging from 0.93 to 0.94, and when compared to Giarmatzis and colleagues^14^ study that reported correlation coefficients ranging from 0.89 to 0.98. However, the previous study included some trials from the participants in the training set and other trials from the same participants in the test set. When the trials from participants were in the training set or the test set, correlation coefficients ranged from 0.45 to 0.85.

In general, our results show that the 1st force peak was accurately predicted, even when only primary kinematic and kinetic data was used as the training dataset. Gaussian regressors and variations (Kernel exponential, matern 32, and matern 52) provided promising results with coefficients of determination above 0.70 and relative peak error under 7%. The Gaussian regressors family is considered a non-parametric model, which considers the probability distribution over all admissible functions that fit the data, allowing for flexible modeling of complex and non-linear relationships between variables^35^. During gait, the 1st tibiofemoral contact force is clinically relevant because it is related to the maximum force experienced by the knee joint during the initial contact of the foot with the ground. This moment is related to quadriceps eccentric contraction to counterbalance knee flexion during the loading response phase. A good prediction of this variable extends the possibility of understanding the knee compressive loads that may represent a magnitude of approximately 3 times body weight at normal walking speed^39^. Our comprehensive evaluation suggests that this information may be accurately predicted with a relative amount of biomechanical data.

On the other hand, ML models needed more information to present good performance to predict the 2nd tibiofemoral contact force peak, mainly when the All participants group was evaluated. For predictions specifically in OA or Control groups, Input 1 and Input 2 datasets were enough. Linear SVR presented the highest accuracy for All participants group. However, it demanded more complex data for good predictions, such as information on muscle forces. For the OA group, promising results were identified for Fast Decorrelated Neural Network Ensembles (DNNE) considering that only Input 1 training data was enough for accurate predictions. DNNE randomly initializes the hidden layer parameters of base random vector functional link networks and then employs the least square method with a negative correlation learning scheme to analytically calculate the output weights of these base networks^40^. It is a fast and efficient solution to build ensemble models, which facilitates its application for analyzing biomechanical data, reducing the computational bottleneck for obtaining internal biomechanical parameters. For the Control group, 2nd tibiofemoral force peak, promising results were obtained with the Quadratic Support Vector Machine model, with Input 2 as the training dataset. Quadradic SVR also performed well in other health science problems (e.g., brain age prediction) showing flexibility in data-generalization^35^. The second tibiofemoral contact force is clinically relevant during gait because it is associated with the push-off phase. This phase is critical for efficient forward movement and may be connected to functional ability. The difficulty in predicting the second peak may be attributed to the different coordination patterns observed during late stance. As demonstrated in Fig. 2, exploratory analysis allows to visualize that the data distribution for the 2nd peak presents great variability, mainly for the OA group. We speculate that this variability may explain the worst predictions of the machine learning models for the 2nd peak.

The most promising results were achieved when the OA and Control participants were tested separately. This indicates that using models according to participants’ diagnoses/characteristics may improve the model’s output. Table 11 provides a summary of the best model for each group based on the following criteria: the need for the least amount of data as input (i.e., Input 1 is preferred over Input 2 and Input 2 over Input 3), the model with the lowest MAE but with at least 0.7 variance explained^36,37^. The models may be chosen accordingly if a participant is properly classified between knee OA or healthy. If there is no clear classification the participant may be evaluated as belonging to the ‘All participants’ group. For these situations, it may be necessary to collect all variables included in Input 3 to a more accurate prediction of the 2nd tibiofemoral force peak during gait. However, in terms hardware or computational bottlenecks avoidance, Input 3 represents almost the entire process of data processing and analysis, including the long-lasting static optimization procedure. One can argue that there is no great advantages in using Input 3 to reduce the associated processing time. In this sense, it important to emphasize that when Input 3 was used as training dataset, RPE was around 4.7%, against $\approx$10% for the Quadratic SVR when Input 2 was used. Thus, researchers and clinic professionals may evaluate the pros and cons of every model and input combinations to choose the most appropriate procedure depending on the evaluation objectives and assumed error thresholds. Additionally, it seems promising to perform an in-depth evaluation regarding the roles that each variable presents on the predictions quality. Although an evaluation of the weights of each variable on the model is possible for the linear models, the non-linear models are more complex and requires further development of new algorithms to identify the key-variables and the explained variance for the predictions with the best models.

Future work may investigate, from evaluating these 24 models, a fusion^41^ of the best-performed ones for even improved accuracy prediction with the least amount of data required. Additionally, the promising results of tibiofemoral contact forces estimate from primary kinematic and kinetic data highlight a broad possibility of providing accurate biomechanical analysis in clinical settings. More than that, IMU^24^ and markerless systems^42^ represent low-cost alternatives to provide biomechanical data that, together with ML algorithms evaluated in the present study, may supply joint contact forces reports with very low time-consuming protocols.

**Table 11:** Summary of the best algorithms for each group.

Group	1st Knee contact peak	2nd Knee contact peak
All patients	Input1 + Gaussian regression (Kernel-exponential)	Input3 + Linear SVR
OA patients	Input1 + Gaussian regression (Kernel-matern 32)	Input1 + DNNE
Control	Input1 + Gaussian regression (Kernel-matern 52)	Input2 + Quadratic SVR

Finally, this study has some limitations to be highlighted. Although our sample size is relatively large considering the specific inclusion criteria for the OA patients, larger datasets are desirable for ML evaluation study design. Thus, it is possible that, with more samples, other ML models may outperform the ones reported in the present study, or even better accurate the predictions achieved with the best models presented here. Also, the symptomatic group was composed of severe unilateral knee OA (KL4 class). Thus, our results may not be generalized for different stages of OA. Further studies concerning the assessment of ML methods in scenarios in which more variation in the OA characteristics between patients can be included in both training and test datasets will help to improve model’s prediction. In this sense, two alternatives deserve attention. The first one is that machine learning models may benefit from public multimodal datasets^43^ to improve the training step. However, it is also necessary a cooperation from the scientific community to provide public datasets not only of injury-free participants but pathological individuals, such as OA patients. The second promising alternative is to develop deep learning (DL) solutions presented in literature for synthetic data generation, such as “generative adversarial networks” (GANs)^44^. Future studies may also investigate the potential of such data augmentation strategies to improve the accuracy of the models, specifically for pathological individuals in respect to their physical function condition. We also emphasize that both the training and test dataset included males and females. One can argue that sex-specific regression models may outperform generic models. However, an additional split in our data for female and male dataset training and testing would restrict the generalization of the results. On the other hand, considering the very promising results reported in the present study with a joined sample, future studies with refined models is highly recommended. Lastly, it is important to consider that the tibiofemoral forces results used in this study are derived from musculoskeletal simulations, and the outcomes are influenced by factors such as the choice of the model, scaling techniques, and optimization processes^10,45^. However, it should be noted that direct *in vivo* measurements have limitations in terms of sample size and their applicability, as they rely on the use of an instrumented knee prosthesis.

## Conclusion

This study evaluated 24 machine learning models to predict tibiofemoral contact forces in healthy individuals and knee OA patients. Machine learning models could predict tibiofemoral contact forces, and may be an alternative for sites with fewer structures for biomechanical evaluations. Our study provided insights into the most promising models considering the amount of biomechanical data required as input data according to participant’s classification (healthy or knee OA), representing an important starting point for the generalization of biomechanical analyses in clinical settings, as well as for improvements in musculoskeletal models equations for the calculation of joint reaction forces.

### Supplementary Information

Supplementary Tables.

## Data Availability

The MSK model developed in OpenSim and employed in this study is freely available for download at https://simtk.org/projects/tcf_comp_forces (As of Dec. 2023). All the additional data is provided in the manuscript and appendices.
