# Supplementary material for: On the prediction of tibiofemoral contact forces for healthy individuals and osteoarthritis patients during gait: a comparative study of regression methods
Source: Sci Rep. 2024 Jan 16;14:1379. doi: 10.1038/s41598-023-50481-x (PMC10791669; doi:10.1038/s41598-023-50481-x)
Supplement: Supplementary file 1 — Supplementary Tables. [file 41598_2023_50481_MOESM1_ESM.pdf]

Appendix A Hyperparameter Selection

Table 12: The parameters and hyperparameters tuning (when applicable) tested and selected over experiments (considering the input data), and respective references for each algorithm.

**Function**	**Parameters / Hyperparameters tested**	**Input 1 - Parameters / Hyperparameters selected**	**Input 2 - Parameters /Hyperparameters selected**	**Input 3 - Parameters /Hyperparameters selected**	**Ref.**
1) Ensemble Trees (Bagging)	Gridsearch for learning cycles	1st Peak: 160 learning cycles, cross-validation off	1st Peak: 160 learning cycles, cross-validation off	1st Peak: 160 learning cycles, leave-one-out cross-validation	[46]
	Cross-validation (off, leave-one-out)	2nd Peak: 160 learning cycles, leave-one-out cross-validation	2nd Peak: 160 learning cycles, leave-one-out cross-validation	2nd Peak: 160 learning cycles, leave-one-out cross-validation	
2) Ensemble Trees (LSBoost)	Gridsearch for learning cycles	1st Peak: 160 learning cycles, cross-validation off	1st Peak: 160 learning cycles, cross-validation off	1st Peak: 160 learning cycles, cross-validation off	[46]
	Cross-validation (off, leave-one-out)	2nd Peak: 160 learning cycles, leave-one-out cross-validation	2nd Peak: 160 learning cycles, cross-validation off	2nd Peak: 160 learning cycles, cross-validation off	
3) Linear Support Vector Machine (SVR)	Standardize (on/off)	1st Peak: Standardize off, leave-one-out cross-validation, Hyperparameters tuning off	1st Peak: Standardize off, leave-one-out cross-validation, Hyperparameters tuning off	1st Peak: Standardize off, Hyperparameters tuning = probability of improvement	[47, 48]
	Cross-validation (off, leave-one-out)	2nd Peak: Standardize off, Hyperparameters tuning = gridsearch	2nd Peak: Standardize off, Hyperparameters tuning = expected-improvement-per-second-plus	2nd Peak: Standardize off, Hyperparameters tuning = expected-improvement-plus	
	Hyperparameters tuning (off, Bayesian optimization classes [Expected Improvement, Probability of Improvement, Lower Confidence Bound,Per Second, Plus], gridsearch, randomsearch)				
4) Quadratic SVR	Standardize (on/off)	1st Peak: Standardize off, Hyperparameters tuning = probability-of-improvement	1st Peak: Standardize off, Hyperparameters tuning = expected-improvement-plus	1st Peak: Standardize off, Hyperparameters tuning = expected-improvement-per-second-plus	[47, 48]
	Cross-validation (off, leave-one-out)	2nd Peak: Standardize on, Hyperparameters tuning = expected-improvement	2nd Peak: Standardize on, Hyperparameters tuning = expected-improvement-per-second-plus	2nd Peak: Standardize on, Hyperparameters tuning = lower confidence bound	
	Hyperparameters tuning (off, Bayesian optimization classes [Expected Improvement, Probability of Improvement, Lower Confidence Bound,Per Second, Plus], gridsearch, randomsearch)				
5) Cubic SVR	Standardize (on/off)	1st Peak: Standardize on, Hyperparameters tuning = randomsearch	1st Peak: Standardize on, Hyperparameters tuning = expected-improvement-per-second-plus	1st Peak: Standardize on, Hyperparameters tuning = expected-improvement-per-second-plus	[47, 48]
	Cross-validation (off, leave-one-out)	2nd Peak: Standardize on, Hyperparameters tuning = randomsearch	2nd Peak: Standardize on, Hyperparameters tuning = expected-improvement-per-second	2nd Peak: Standardize on, Hyperparameters tuning = expected-improvement-per-second-plus	

Table 12: The parameters and hyperparameters tuning (when applicable) tested and selected over experiments (considering the input data), and respective references for each algorithm (Continued).

Function	Parameters / Hyperparameters tested	Input 1 - Parameters / Hyperparameters selected	Input 2 - Parameters /Hyperparameters selected	Input 3 - Parameters /Hyperparameters selected	Ref.
	Hyperparameters tuning (off, Bayesian optimization classes [Expected Improvement, Probability of Improvement, Lower Confidence Bound, Per Second, Plus], gridsearch, randomsearch)				
6) Gaussian SVR	Standardize (on/off)	1st Peak: Standardize off, Hyperparameters tuning = expected-improvement-per-second	1st Peak: Standardize off, Hyperparameters tuning = expected-improvement	1st Peak: Standardize on, Hyperparameters tuning = lower-confidence-bound	[47, 48]
	Cross-validation (off, leave-one-out)	2nd Peak: Standardize on, Hyperparameters tuning = expected-improvement	2nd Peak: Standardize on, Hyperparameters tuning = gridsearch	2nd Peak: Standardize on, Hyperparameters tuning = gridsearch	
	Hyperparameters tuning (off, Bayesian optimization classes [Expected Improvement, Probability of Improvement, Lower Confidence Bound, Per Second, Plus], gridsearch, randomsearch)				
7) Linear Regression	Cross-validation (off, leave-one-out)	1st Peak: leave-one-out cross-validation	1st Peak: leave-one-out cross-validation	1st Peak: leave-one-out cross-validation	[36]
		2nd Peak: leave-one-out cross-validation	2nd Peak: leave-one-out cross-validation	2nd Peak: leave-one-out cross-validation	
8) Lasso Regression	Cross-validation (leave-one-out)	1st Peak: leave-one-out cross-validation, alpha = 0.75	1st Peak: leave-one-out cross-validation, alpha = 0.75	1st Peak: leave-one-out cross-validation, alpha = 0.75	[49]
	Elastic net (alpha = 0.5, 0.75 or 0.8)	2nd Peak: leave-one-out cross-validation, alpha = 0.75	2nd Peak: leave-one-out cross-validation, alpha = 0.75	2nd Peak: leave-one-out cross-validation, alpha = 0.75	
9) Ridge Regression	Cross-validation (leave-one-out)	1st Peak: leave-one-out cross-validation, k = 5	1st Peak: leave-one-out cross-validation, k = 3	1st Peak: leave-one-out cross-validation, k = 1	[50]
	Ridge parameters (k = [1,5])	2nd Peak: leave-one-out cross-validation, k = 1	2nd Peak: leave-one-out cross-validation, k = 1	2nd Peak: leave-one-out cross-validation, k = 5	
10) Binary Decision Tree	Cross-validation (off, leave-one-out)	1st Peak: Hyperparameters tuning = randomsearch, MinLeafSize = 2	1st Peak: Hyperparameters tuning = expected-improvement-plus, MinLeafSize = 1	1st Peak: leave-one-out cross-validation, MinLeafSize = 1	[51]
	Minimal leaf size (MinLeafSize) observations Hyperparameters tuning for (off, Bayesian optimization classes [Expected Improvement, Probability of Improvement, Lower Confidence Bound, Per Second, Plus], gridsearch, randomsearch)	2nd Peak: Hyperparameters tuning = randomsearch, MinLeafSize = 4	2nd Peak: Hyperparameters tuning = gridsearch, MinLeafSize = 13	2nd Peak: Hyperparameters tuning = randomsearch, MinLeafSize = 13	
11) Gaussian Regression (Kernel - exponential)	Standardize (on/off)	1st Peak: Standardize off, leave-one-out cross-validation	1st Peak: Standardize off, leave-one-out cross-validation	1st Peak: Standardize on, leave-one-out cross-validation	[52]

Table 12: The parameters and hyperparameters tuning (when applicable) tested and selected over experiments (considering the input data), and respective references for each algorithm (Continued).

Function	Parameters / Hyperparameters tested	Input 1 - Parameters / Hyperparameters selected	Input 2 - Parameters /Hyperparameters selected	Input 3 - Parameters /Hyperparameters selected	Ref.
	Cross-validation (off, leave-one-out)	2nd Peak: Standardize on, leave-one-out cross-validation	2nd Peak: Standardize on, Hyperparameters tuning = lower-confidence-bound,	2nd Peak: Standardize on, leave-one-out cross-validation	
	Hyperparameters tuning (off, Bayesian optimization classes [Expected Improvement, Probability of Improvement, Lower Confidence Bound, Per Second, Plus], gridsearch, randomsearch)				
12) Gaussian Regression (Kernel - squared exponential)	Standardize (on/off)	1st Peak: Standardize off, leave-one-out cross-validation	1st Peak: Standardize on, leave-one-out cross-validation	1st Peak: Standardize on, Hyperparameters tuning = expected-improvement-per-second-plus	[52]
	Cross-validation (off, leave-one-out)	2nd Peak: Standardize on, leave-one-out cross-validation	2nd Peak: Standardize on, leave-one-out cross-validation	2nd Peak: Standardize on, leave-one-out cross-validation	
	Hyperparameters tuning (off, Bayesian optimization classes [Expected Improvement, Probability of Improvement, Lower Confidence Bound, Per Second, Plus], gridsearch, randomsearch)				
13) Gaussian Regression (Kernel - matern 32)	Standardize (on/off)	1st Peak: Standardize on, Hyperparameters tuning = expected-improvement-per-second-plus	1st Peak: Standardize off, leave-one-out cross-validation	1st Peak: Standardize on, Hyperparameters tuning = expected-improvement-per-second-plus	[52]
	Cross-validation (off, leave-one-out)	2nd Peak: Standardize on, Hyperparameters tuning = gridsearch	2nd Peak: Standardize on, Hyperparameters tuning = lower-confidence-bound	2nd Peak: Standardize on, Hyperparameters tuning = expected-improvement-per-second-plus	
	Hyperparameters tuning (off, Bayesian optimization classes [Expected Improvement, Probability of Improvement, Lower Confidence Bound, Per Second, Plus], gridsearch, randomsearch)				
14) Gaussian Regression (Kernel - matern 52)	Standardize (on/off)	1st Peak: Standardize off, leave-one-out cross-validation	1st Peak: Standardize off, leave-one-out cross-validation	1st Peak: Standardize on, leave-one-out cross-validation	[52]
	Cross-validation (off, leave-one-out)	2nd Peak: Standardize on, leave-one-out cross-validation	2nd Peak: Standardize on, leave-one-out cross-validation	2nd Peak: Standardize off, leave-one-out cross-validation	
	Hyperparameters tuning (off, Bayesian optimization classes [Expected Improvement, Probability of Improvement, Lower Confidence Bound, Per Second, Plus], gridsearch, randomsearch)				

Table 12: The parameters and hyperparameters tuning (when applicable) tested and selected over experiments (considering the input data), and respective references for each algorithm (Continued).

Function	Parameters / Hyperparameters tested	Input 1 - Parameters / Hyperparameters selected	Input 2 - Parameters /Hyperparameters selected	Input 3 - Parameters /Hyperparameters selected	Ref.
15) Gaussian Regression (Kernel - rational quadratic)	Standardize (on/off)	1st Peak: Standardize off, leave-one-out cross-validation	1st Peak: Standardize off, leave-one-out cross-validation	1st Peak: Standardize on, Hyperparameters tuning = expected-improvement-per-second-plus	[52]
	Cross-validation (off, leave-one-out)	2nd Peak: Standardize on, leave-one-out cross-validation	2nd Peak: Standardize on, leave-one-out cross-validation	2nd Peak: Standardize on, leave-one-out cross-validation	
	Hyperparameters tuning (off, Bayesian optimization classes [Expected Improvement, Probability of Improvement, Lower Confidence Bound, Per Second, Plus], gridsearch, randomsearch)				
16) Epsilon Twin Support Vector Regression (ETSVR)	Cross-validation (10-fold)	1st Peak: Fixed options	1st Peak: Fixed options	1st Peak: Fixed options	[53]
	Hyperparameters tuning (grisearch of $[2^{-8}, 2^{-8}]$)	2nd Peak: Fixed options	2nd Peak: Fixed options	2nd Peak: Fixed options	
17) Kernel Ridge Regression	Cross-validation (10-fold)	1st Peak: Fixed options	1st Peak: Fixed options	1st Peak: Fixed options	[54]
	Hyperparameters tuning(alpha regularization $[2^{-5}, 2^{10}]$)	2nd Peak: Fixed options	2nd Peak: Fixed options	2nd Peak: Fixed options	
18) Nyström Ridge Regression	Cross-validation (10-fold)	1st Peak: Fixed options	1st Peak: Fixed options	1st Peak: Fixed options	[55]
	Hyperparameters tuning(alpha regularization $[2^{-5}, 2^{10}]$)	2nd Peak: Fixed options	2nd Peak: Fixed options	2nd Peak: Fixed options	
19) DNNE	Cross-validation (10-fold)	1st Peak: Ensemble size = 150 , Number of basis functions = 6, boosting threshold = 0.6	1st Peak: Ensemble size = 100 , Number of basis functions = 6, boosting threshold = 0.6	1st Peak: Ensemble size = 220 , Number of basis functions = 6, boosting threshold = 0.6	
	Ensemble size ([2,15], step by 1)	2nd Peak:Ensemble size = 150 , Number of basis functions = 6, boosting threshold = 0.6	2nd Peak:Ensemble size = 100 , Number of basis functions = 6, boosting threshold = 0.6	2nd Peak:Ensemble size = 100 , Number of basis functions = 6, boosting threshold = 0.6	[40]
	Number of basis functions ([5,200], step by 5)				
	Boosting threshold ([0.1,1], step by 0.1				
20) kNN Weighted Mean	Cross-validation (10-fold)	1st Peak: k = 3	1st Peak: k = 3	1st Peak: k = 3	[56]
	K-neighbors ([1,10])	2nd Peak: k = 2	2nd Peak: k = 2	2nd Peak: k = 2	
21) Regularized K-Nearest Neighbor based Weighted Twin Support Vector Regression (RKNNWTSVR)	Cross-validation (10-fold)	1st Peak: p1 = 10^{-1}, p2 = 10^{5}	1st Peak: p1 = 10^{1}, p2 = 10^{4}	1st Peak: p1 = 10^{-1}, p2 = 10^{5}	[57]
	Parameter p1 ($[10^{-5}, 10^{5}]$)	2nd Peak: p1 = 10^{-5}, p2 = 10^{5}	2nd Peak: p1 = 10^{-5}, p2 = 10^{-1}	2nd Peak: p1 = 10^{-3}, p2 = 10^{5}	
	Parameter p2 ($[10^{-5}, 10^{5}]$)				
22) Lagrangian Twin Support Vector Regression (LTSVR)	Cross-validation (10-fold)	1st Peak: p1 = 10^{-5}	1st Peak: p1 = 10^{-5}	1st Peak: p1 = 10^{-1}	[58]
	Parameter p1 ($[10^{-5}, 10^{5}]$)	2nd Peak: p1 = 10^{-5}	2nd Peak: p1 = 10^{-5}	2nd Peak: p1 = 10^{-4}	
23) Stepwise glm	Distribution of the response variable (normal, binomial, poisson, gamma, inverse gaussian)	1st Peak: Poisson and Akaike information criterion	1st Peak: Poisson and Akaike information criterion	1st Peak: Poisson and Akaike information criterion	[59]

Table 12: The parameters and hyperparameters tuning (when applicable) tested and selected over experiments (considering the input data), and respective references for each algorithm (Continued).

Function	Parameters / Hyperparameters tested	Input 1 - Parameters / Hyperparameters selected	Input 2 - Parameters /Hyperparameters selected	Input 3 - Parameters /Hyperparameters selected	Ref.
	Criterion to add or remove terms	2nd Peak: Poisson and Akaike information criterion	2nd Peak: Poisson and Akaike information criterion	2nd Peak: Poisson and Akaike information criterion	
24) Neural Networks	Cross-validation (10-fold)<br>Epochs size([5:5:100])<br>Number of layers([2:15])	1st Peak: layers = 4, epochs = 90<br>2nd Peak: layers = 6, epochs = 195	1st Peak: layers = 4, epochs = 80<br>2nd Peak: layers = 4, epochs = 20	1st Peak: layers = 4, epochs = 100<br>2nd Peak: layers = 8, epochs = 100	[60]

Appendix B Training Results

Table 13: Summary of the algorithms training performance for Input 1 data.

Function	1st Knee contact peak (N/body weight)								2nd Knee contact peak (N/body weight)							
	MAE	RPE	RMSE	R	R2	MDF	LCI	UCI	MAE	RPE	RMSE	R	R2	MDF	LCI	UCI
1) Ensemble Trees (Bagging)	0.10	3.68	0.13	0.98	0.95	0.00	-0.03	0.02	0.12	3.57	0.17	0.95	0.90	-0.01	-0.04	0.03
2) Ensemble Trees (LSBoost)	0.01	0.54	0.02	1.00	1.00	0.00	0.00	0.00	0.02	0.50	0.02	1.00	1.00	0.00	0.00	0.00
3) Linear SVR	0.16	6.32	0.21	0.93	0.86	0.01	-0.04	0.05	0.23	7.25	0.31	0.80	0.64	0.00	-0.07	0.06
4) Quadratic SVR	0.12	4.30	0.14	0.97	0.94	-0.01	-0.04	0.02	0.16	4.75	0.25	0.87	0.75	-0.02	-0.07	0.03
5) Cubic SVR	0.17	6.45	0.20	0.94	0.89	-0.05	-0.09	-0.01	0.01	0.41	0.01	1.00	1.00	0.00	0.00	0.00
6) Gaussian SVR	0.06	2.27	0.11	0.98	0.96	-0.01	-0.03	0.01	0.03	0.86	0.13	0.97	0.94	-0.01	-0.04	0.01
7) Linear Regression	0.22	8.57	0.30	0.85	0.72	0.04	-0.02	0.10	0.24	7.69	0.34	0.74	0.55	0.00	-0.07	0.08
8) Lasso Regression	0.16	6.05	0.19	0.94	0.89	0.00	-0.04	0.04	0.25	7.97	0.33	0.78	0.61	0.00	-0.07	0.07
9) Ridge Regression	0.14	5.36	0.17	0.95	0.90	0.00	-0.04	0.04	0.21	6.74	0.29	0.83	0.68	0.00	-0.06	0.06
10) Binary Decision Tree	0.09	3.21	0.12	0.98	0.96	0.00	-0.02	0.02	0.12	3.64	0.16	0.95	0.90	0.00	-0.03	0.03
11) GR (K. - exponential)	0.00	0.00	0.00	1.00	1.00	0.00	0.00	0.00	0.00	0.00	0.00	1.00	1.00	0.00	0.00	0.00
12) GR (K. - squared exponential)	0.06	2.03	0.07	0.99	0.98	0.00	-0.01	0.02	0.00	0.00	0.00	1.00	1.00	0.00	0.00	0.00
13) GR (K. - matern 32)	0.05	2.04	0.07	0.99	0.99	0.00	-0.02	0.02	0.00	0.00	0.00	1.00	1.00	0.00	0.00	0.00
14) GR (K. - matern 52)	0.04	1.61	0.06	0.99	0.99	0.00	-0.01	0.02	0.00	0.00	0.00	1.00	1.00	0.00	0.00	0.00
15) GR (K. - rational quadratic)	0.05	1.67	0.06	0.99	0.99	0.00	-0.01	0.02	0.00	0.00	0.00	1.00	1.00	0.00	0.00	0.00
16) ETSVR - Kernel Linear	0.14	5.41	0.17	0.95	0.90	0.00	-0.04	0.04	0.22	6.76	0.29	0.82	0.68	-0.06	-0.12	0.01
17) Kernel Ridge Regression	0.14	5.55	0.18	0.95	0.90	0.00	-0.03	0.04	0.22	6.87	0.29	0.83	0.68	0.01	-0.05	0.07
18) Nyström Ridge Regression	0.14	5.47	0.17	0.95	0.90	0.00	-0.03	0.04	0.22	6.81	0.29	0.83	0.68	0.01	-0.05	0.07
19) DNNE	0.00	0.00	0.00	1.00	1.00	0.00	0.00	0.00	0.00	0.00	0.00	1.00	1.00	0.00	0.00	0.00
20) kNN Weghted Mean	0.10	3.88	0.16	0.96	0.92	0.00	-0.03	0.04	0.08	2.22	0.12	0.97	0.95	-0.01	-0.03	0.02
21) RKNNWTSVR	0.15	5.61	0.18	0.95	0.90	-0.01	-0.05	0.03	0.22	7.06	0.30	0.81	0.65	-0.03	-0.09	0.04
22) LTSVR	0.14	5.28	0.17	0.95	0.91	0.00	-0.04	0.04	0.21	6.71	0.29	0.83	0.68	0.00	-0.06	0.06
23) Stepwise glm	0.21	7.80	0.25	0.90	0.81	0.00	-0.05	0.05	0.34	10.76	0.42	0.56	0.31	0.00	-0.09	0.09
24) Neural Networks	0.10	3.85	0.14	0.97	0.94	-0.01	-0.04	0.02	0.25	8.00	0.32	0.79	0.62	0.01	-0.06	0.08

RMSE: root mean squared error, R: Pearson Correlation Coefficient, (R^2): the coefficient of determination, MDF: Mean Delta Force, LCI: Lower confidence interval, UPF: Upper confidence interval, GR: Gaussian Regression, K: Kernel.

Table 14: Summary of the algorithms training performance for Input 2 data.

Function	1st Knee contact peak (N/body weight)								2nd Knee contact peak (N/body weight)							
	MAE	RPE	RMSE	R	R2	MDF	LCI	UCI	MAE	RPE	RMSE	R	R2	MDF	LCI	UCI
1) Ensemble Trees (Bagging)	0.09	3.29	0.13	0.98	0.95	0.00	-0.03	0.03	0.11	3.52	0.17	0.95	0.90	0.00	-0.04	0.03
2) Ensemble Trees (LSBoost)	0.01	0.28	0.01	1.00	1.00	0.00	0.00	0.00	0.01	0.27	0.01	1.00	1.00	0.00	0.00	0.00
3) Linear SVR	0.15	6.08	0.20	0.93	0.87	0.02	-0.03	0.06	0.17	5.24	0.26	0.86	0.75	0.00	-0.06	0.05
4) Quadratic SVR	0.07	2.43	0.08	0.99	0.98	0.00	-0.02	0.01	0.03	1.19	0.04	1.00	1.00	0.00	-0.01	0.01
5) Cubic SVR	0.05	1.76	0.08	0.99	0.98	0.00	-0.02	0.02	0.00	0.02	0.00	1.00	1.00	0.00	0.00	0.00
6) Gaussian SVR	0.07	2.52	0.12	0.98	0.96	-0.01	-0.03	0.02	0.06	1.94	0.07	0.99	0.98	0.01	0.00	0.03
7) Linear Regression	0.22	8.59	0.30	0.85	0.73	0.04	-0.02	0.11	0.22	7.06	0.32	0.78	0.61	0.01	-0.05	0.08
8) Lasso Regression	0.13	4.81	0.17	0.96	0.92	0.00	-0.04	0.04	0.19	5.98	0.25	0.88	0.77	0.00	-0.05	0.05
9) Ridge Regression	0.11	4.28	0.14	0.97	0.93	0.00	-0.03	0.03	0.16	5.11	0.21	0.91	0.82	0.00	-0.05	0.05
10) Binary Decision Tree	0.06	2.20	0.09	0.99	0.98	0.00	-0.02	0.02	0.20	6.14	0.27	0.85	0.73	0.00	-0.06	0.06
11) GR (K. - exponential)	0.06	2.03	0.07	0.99	0.98	0.00	-0.01	0.02	0.00	0.00	0.00	1.00	1.00	0.00	0.00	0.00
12) GR (K. - squared exponential)	0.00	0.01	0.00	1.00	1.00	0.00	0.00	0.00	0.00	0.01	0.00	1.00	1.00	0.00	0.00	0.00
13) GR (K. - matern 32)	0.04	1.31	0.05	1.00	0.99	0.00	-0.01	0.01	0.05	1.37	0.06	0.99	0.99	0.00	-0.01	0.01
14) GR (K. - matern 52)	0.04	1.61	0.06	0.99	0.99	0.00	-0.01	0.02	0.00	0.00	0.00	1.00	1.00	0.00	0.00	0.00
15) GR (K. - rational quadratic)	0.05	1.67	0.06	0.99	0.99	0.00	-0.01	0.02	0.00	0.00	0.00	1.00	1.00	0.00	0.00	0.00
16) ETSVR - Kernel Linear	0.12	4.41	0.15	0.97	0.93	0.00	-0.03	0.03	0.17	5.18	0.23	0.90	0.81	-0.04	-0.09	0.01
17) Kernel Ridge Regression	0.11	4.29	0.14	0.97	0.93	0.01	-0.02	0.04	0.17	5.37	0.23	0.90	0.80	0.01	-0.04	0.06
18) Nyström Ridge Regression	0.13	4.84	0.16	0.96	0.91	0.01	-0.03	0.04	0.25	7.90	0.32	0.82	0.68	0.02	-0.05	0.08
19) DNNE	0.00	0.00	0.00	1.00	1.00	0.00	0.00	0.00	0.00	0.00	0.00	1.00	1.00	0.00	0.00	0.00
20) kNN Weghted Mean	0.10	3.90	0.16	0.96	0.92	0.00	-0.03	0.04	0.08	2.25	0.12	0.97	0.94	-0.01	-0.03	0.02
21) RKNNWTSVR	0.14	5.25	0.17	0.95	0.91	-0.02	-0.05	0.02	0.19	5.93	0.26	0.86	0.74	-0.02	-0.08	0.03
22) LTSVR	0.11	4.10	0.14	0.97	0.94	0.00	-0.03	0.03	0.17	5.16	0.21	0.91	0.83	0.00	-0.05	0.05
23) Stepwise glm	0.16	5.84	0.25	0.89	0.80	0.00	-0.05	0.05	0.34	10.76	0.42	0.56	0.31	0.00	-0.09	0.09
24) Neural Networks	0.09	3.19	0.11	0.98	0.96	0.00	-0.03	0.02	0.25	7.71	0.33	0.77	0.59	0.00	-0.07	0.07

RMSE: root mean squared error, R: Pearson Correlation Coefficient, (R^2): the coefficient of determination, MDF: Mean Delta Force, LCI: Lower confidence interval, UPF: Upper confidence interval, GR: Gaussian Regression, K: Kernel.

Table 15: Summary of the algorithms training performance for Input 3 data.

Function	1st Knee contact peak (N/body weight)								2nd Knee contact peak (N/body weight)							
	MAE	RPE	RMSE	R	R2	MDF	LCI	UCI	MAE	RPE	RMSE	R	R2	MDF	LCI	UCI
1) Ensemble Trees (Bagging)	0.07	2.61	0.09	0.99	0.97	0.00	-0.02	0.02	0.09	2.77	0.13	0.97	0.95	0.00	-0.02	0.03
2) Ensemble Trees (LSBoost)	0.00	0.12	0.00	1.00	1.00	0.00	0.00	0.00	0.00	0.09	0.00	1.00	1.00	0.00	0.00	0.00
3) Linear SVR	0.04	1.50	0.08	0.99	0.98	0.01	-0.01	0.02	0.04	1.33	0.08	0.99	0.98	0.00	-0.02	0.01
4) Quadratic SVR	0.03	1.32	0.06	0.99	0.99	0.00	-0.01	0.02	0.00	0.02	0.00	1.00	1.00	0.00	0.00	0.00
5) Cubic SVR	0.04	1.39	0.07	0.99	0.99	0.00	-0.01	0.02	0.01	0.15	0.01	1.00	1.00	0.00	0.00	0.00
6) Gaussian SVR	0.01	0.39	0.03	1.00	1.00	0.00	0.00	0.01	0.00	0.02	0.00	1.00	1.00	0.00	0.00	0.00
7) Linear Regression	0.13	5.25	0.19	0.94	0.89	0.02	-0.02	0.06	0.21	6.53	0.27	0.85	0.72	0.00	-0.06	0.06
8) Lasso Regression	0.09	3.35	0.12	0.98	0.96	0.00	-0.03	0.03	0.12	3.74	0.14	0.97	0.95	0.00	-0.03	0.03
9) Ridge Regression	0.05	1.75	0.06	0.99	0.99	0.00	-0.01	0.01	0.04	1.28	0.06	0.99	0.99	0.00	-0.01	0.01
10) Binary Decision Tree	0.06	2.13	0.08	0.99	0.98	0.00	-0.02	0.02	0.07	2.12	0.11	0.98	0.96	0.00	-0.02	0.02
11) GR (K. - exponential)	0.00	0.00	0.00	1.00	1.00	0.00	0.00	0.00	0.00	0.00	0.00	1.00	1.00	0.00	0.00	0.00
12) GR (K. - squared exponential)	0.02	0.94	0.03	1.00	1.00	0.00	-0.01	0.01	0.00	0.01	0.00	1.00	1.00	0.00	0.00	0.00
13) GR (K. - matern 32)	0.01	0.24	0.01	1.00	1.00	0.00	0.00	0.00	0.00	0.01	0.00	1.00	1.00	0.00	0.00	0.00
14) GR (K. - matern 52)	0.00	0.02	0.00	1.00	1.00	0.00	0.00	0.00	0.00	0.01	0.00	1.00	1.00	0.00	0.00	0.00
15) GR (K. - rational quadratic)	0.02	0.74	0.03	1.00	1.00	0.00	-0.01	0.01	0.00	0.01	0.00	1.00	1.00	0.00	0.00	0.00
16) ETSVR - Kernel Linear	0.04	1.72	0.06	0.99	0.99	0.01	-0.01	0.02	0.04	1.27	0.05	1.00	0.99	0.00	-0.01	0.01
17) Kernel Ridge Regression	0.05	1.79	0.06	0.99	0.99	0.00	-0.01	0.01	0.04	1.22	0.05	1.00	0.99	0.00	-0.01	0.01
18) Nyström Ridge Regression	0.06	2.10	0.07	0.99	0.98	0.00	-0.02	0.01	0.05	1.69	0.07	0.99	0.98	0.00	-0.01	0.02
19) DNNE	0.00	0.00	0.00	1.00	1.00	0.00	0.00	0.00	0.00	0.00	0.00	1.00	1.00	0.00	0.00	0.00
20) kNN Weghted Mean	0.10	3.96	0.15	0.96	0.93	0.01	-0.03	0.04	0.06	1.91	0.10	0.98	0.96	-0.02	-0.04	0.00
21) RKNNWTSVR	0.04	1.74	0.07	0.99	0.99	0.00	-0.01	0.02	0.06	1.84	0.08	0.99	0.98	0.00	-0.02	0.01
22) LTSVR	0.04	1.34	0.04	1.00	0.99	0.00	-0.01	0.01	0.03	0.93	0.04	1.00	0.99	0.00	-0.01	0.01
23) Stepwise glm	0.19	7.12	0.22	0.92	0.85	0.00	-0.05	0.05	0.27	8.45	0.32	0.78	0.61	0.00	-0.07	0.07
24) Neural Networks	0.18	6.85	0.23	0.91	0.83	-0.01	-0.06	0.04	0.04	1.35	0.06	0.99	0.99	0.00	-0.01	0.01

RMSE: root mean squared error, R: Pearson Correlation Coefficient, (R^2): the coefficient of determination, MDF: Mean Delta Force, LCI: Lower confidence interval, UPF: Upper confidence interval, GR: Gaussian Regression, K: Kernel.
